# A Detrimental *NFKB2* Missense Variant is Associated with Hypogammaglobulinemia

**DOI:** 10.1007/s10875-026-02051-9

**Published:** 2026-07-11

**Authors:** Manfred Fliegauf, Laura Gamez-Diaz, Pavla Mrovecova, Chiara Milena God, Valerie Flavia Geiger, Nadezhda Camacho-Ordonez, Sara Posadas-Cantera, Cliodhna Murray, Klaus Warnatz, Baerbel Keller, Bodo Grimbacher

**Affiliations:** 1 Institute for Immunodeficiency (IFI), Center for Chronic Immunodeficiency (CCI), Faculty of Medicine, Medical Center - University of Freiburg, University of Freiburg, Breisacherstraße 115, Freiburg, 79106 Germany; 2 Center for Chronic Immunodeficiency (CCI), Faculty of Medicine, Medical Center - University of Freiburg, University of Freiburg, Freiburg, Germany; 3 CIBSS – Centre for Integrative Biological Signalling Studies, Albert-Ludwigs University, Freiburg, Germany; 4 Department of Rheumatology and Clinical Immunology, Faculty of Medicine, Medical Center-University of Freiburg, University of Freiburg, Freiburg, Germany; 5 Institute for Microbiology, Faculty of Medicine, Medical Center - University of Freiburg, University of Freiburg, Freiburg, Germany; 6 Department of Immunology, University Hospital Zurich, Zurich, Switzerland; 7 DZIF – German Center for Infection Research, Satellite Center Freiburg, Freiburg, Germany; 8 RESIST – Cluster of Excellence 2155 to Hanover Medical School, Satellite Center Freiburg, Freiburg, Germany

**Keywords:** NFKB2, Hypogammaglobulinemia, Common Variable immunodeficiency (CVID), NF-kappaB signaling, Primary Immunodeficiency (PID), Inborn errors of immunity (IEI)

## Abstract

*NFKB2* encodes the precursor p100 which undergoes processing to generate the mature NF-κB2 transcription factor subunit p52. Most of the known pathogenic *NFKB2* variants render p100 un-processable and are typically linked to immunodeficiency disorders with antibody deficiency, susceptibility to infections and often autoimmunity. We describe a heterozygous germline *NFKB2* missense variant (c.781C>T; R261W) associated with antibody deficiency and recurrent respiratory tract infections in a German family with incomplete penetrance. Patient-derived cells had reduced p52 levels, indicating protein insufficiency as the primary defect. Characterization of the R261W variant in vitro employing overexpression of EGFP-fused wildtype and mutant p100/p52 in HEK293T cells revealed restricted levels of mutant p100, suggesting unsustainable protein expression while maintenance of mutant p52 was almost precluded, confirming a detrimental protein defect. Enforced p100-processing driven by constitutively active NF-κB inducing kinase (NIK) caused subnuclear aggregation of mutant EGFP-p52 while DNA-binding activity was undetectable. Although ectopically over-expressed EGFP-p52-R261W also formed intra-nuclear aggregates - a previously established diagnostic indicator of protein-decaying *NFKB1* mutations – its DNA-binding ability was not abolished. In summary, the single amino acid substitution R261W in the N-terminal Rel-homology domain of p100/p52 causes protein loss, particularly of the mature subunit p52. This genetic condition is associated with an inheritable form of hypogammaglobulinemia with mild clinical symptoms. We therefore recommend genetic screening for *NFKB2* loss-of-expression variants in mildly affected patients with recurrent respiratory tract infections.

**Supplementary Information:**

The online version contains supplementary material available at 10.1007/s10875-026-02051-9.

## Introduction

Primary antibody deficiencies (PAD) include a spectrum of inborn errors in antibody production. These range from specific antibody deficiency, in which only the generation of specific antigens such as polysaccharide antigens is impaired, to selective immunoglobulin deficiency, characterized by a reduction in specific immunoglobulin isotypes. More severe forms include deficiencies affecting multiple immunoglobulin classes, as seen in common variable immunodeficiency (CVID), which involves reduced levels of IgG and IgA and/or IgM, or agammaglobulinemia, marked by a complete absence of immunoglobulins. Additionally, some forms of antibody deficiency do not fulfill the criteria for any of the PAD described above and are referred to as unspecified hypogammaglobulinemia.

Whereas monogenetic causes for CVID have been identified [1], monogenetic causes for the milder forms of antibody deficiency remain elusive. However, family members with selective IgG deficiency and pathogenic mutations in *NFKB1* have previously been described [2]. This observation led us to investigate sequence variants in genes of the NF-κB signaling network in patients with mild clinical symptoms.

Heterozygous pathogenic variants in *NFKB2* (nuclear factor kappa B subunit 2, *CVID10*) have first been described in patients with CVID and in patients with a combination of CVID and adrenocorticotropic hormone deficiency, a clinical condition known as DAVID-syndrome (deficit in anterior pituitary function and variable immune deficiency) [3–6]. Revisiting the highly variable ‘NFKB2-disease’ phenotypes described in numerous reports since then, revealed a unique early-onset inborn error of immunity (IEI), with antibody deficiency, T cell dysfunction and various autoimmune features [7–11].

*NFKB1* and *NFKB2* encode two components of the NF-κB (nuclear factor kappa-light-chain-enhancer of activated B-cells) signaling pathways, involved in numerous biological processes. The canonical NF-κB1-dependent pathway primarily mediates fast and broad inflammatory responses whereas the non-canonical NF-κB2-dependent pathway regulates lymphoid organ development, B cell maturation including germinal center reactions, T cell differentiation, thymic selection and antiviral immunity [12–17]. Pathogenic variants in *NFKB1* and *NFKB2* collectively account for the largest subgroup of the monogenetic forms of CVID [7–11, 18, 19].

The NF-κB transcription factor family comprises seven proteins. *NFKB1* and *NFKB2* both encode longer cytoplasmic precursor proteins, p105 and p100, which undergo proteolytic processing of their C-terminal halves to generate the smaller active DNA-binding subunits, p50 and p52, respectively. Their hetero-dimerization partners, RelA (p65), RelB, and c-Rel provide the transcription-activation domain. The five transcription factor monomers can potentially assemble up to 15 homo- and heterodimers with unique functions, and share an N-terminal Rel homology domain (RHD) responsible for dimerization, inhibitor interaction, nuclear localization, and DNA-binding [13, 15, 20].

The activity of pre-assembled NF-κB dimers is mainly controlled by cytoplasmic inhibitory proteins (the ‘classical’ IκBs), including the precursors p100 and p105. The interactions are mediated by an Ankyrin repeat domain (ANK), which is common to all IκBs [20–23]. The non-canonical p100 (the protein encoded by *NFKB2*) therefore has a dual role, serving as the precursor of the mature transcription factor subunit p52 (its N-terminal half) while its C-terminal half serves as an IκB [14, 16, 17, 23]. Non-canonical pathway stimulation *via* surface receptors (such as LTβ, CD40 and BAFF receptors) leads to NIK (NF-κB-inducing kinase)-dependent activation of the IκB-kinase-α (IKKα), which mediates phosphorylation of two serine residues (S866 and S870) within the phospho-degron domain near the C-terminus of p100 [14, 17, 24, 25]. This triggers poly-ubiquitination at Lys855 of p100 and the proteasomal degradation of its C-terminal half to release the mature transcription factor subunit p52 [13–15, 17] with subsequent translocation of dimeric transcription factors (mainly p52/RelB) to the nucleus and target gene regulation.

The vast majority of the known pathogenic *NFKB2* variants cause heterozygous genetic lesions of the C-terminal phosphorylation/ubiquitination domain (degron) of p100 [7–11]. Consequently, since the mutant cytoplasmic p100 precursor persists in a non-processable form, the pathogenic effect originates from both, an excess of the IκB-like activity and the simultaneous shortage of p52 [4, 5, 26, 27]. Less-frequently, heterozygous truncating mutations affecting the central protein domains of p100 predict a bypass of the precursor stage with immediate expression (if expressed at all) of p52-like proteins [7–11, 28–31]. Since the inhibitory activity mediated by p100 is reduced, such variants have been suggested to cause an overall gain-of-NF-κB signaling. In a recent report, a severely truncating variant has been shown to cause haploinsufficiency of both p100 and p52 [32]. While detrimental protein defects due to missense variants within the Rel-homology domain of the canonical p105/p50 are frequent cause of CVID-related diseases [33–35], no such variants have been characterized in *NFKB2* yet.

In the present study, we report a pathogenic *NFKB2* missense variant within the Rel-homology domain of p100/p52 that renders p100 unsustainable and causes a severe p52 defect associated with protein loss. The heterozygous loss of p52 is associated with a mild infection-only phenotype with incomplete penetrance in a German family with IgG and IgM deficiency. Our observations suggest that, unlike the p100 phospho-degron mutations, heterozygous loss-of-expression variants in *NFKB2* may result in only mild clinical symptoms and may therefore be underdiagnosed.

Based on our findings, we recommend to re-analyze *NFKB2* missense variants of uncertain significance (VUS), which have previously been identified upon genetic testing, for functional defects, particularly when causality remains to be assessed in patients with rather mild immunological or infectious disease phenotypes.

## Methods

### Ethical Considerations

This study was carried out in accordance with the recommendations for studies with human subjects of the scientific committee at the University Medical Center of Freiburg. All physicians confirmed that their patients had signed an informed consent under local ethics-approved protocols and in accordance with the Declaration of Helsinki. The study protocol was approved by the ethics committee of the Albert Ludwigs University Freiburg (Approval No. 295/13_200149 and 93/18_191111). No financial incentive was provided, neither to the patients nor the contributing physicians. Data was reported pseudo-anonymized, and physician-to-physician contact allowed to communicate treatment results and advice. Written informed consent was obtained from the participants of the study for the publication of any potentially identifiable data or images in this article.

### Genetic Testing and Mutational Analyses

Genetic analysis was performed by targeted and whole exome next generation sequencing as previously described [36, 37]. The identified sequence changes were subsequently confirmed by Sanger sequencing in the index patient and additional family members according to standard protocols.

### Cell Culture and Transfection

HEK293T cells (human embryonic kidney) were grown in Dulbecco`s modified Eagle medium (DMEM) supplemented with 10% fetal bovine serum (FBS) and 1% penicillin/streptomycin (all from Thermo Fisher Scientific, Karlsruhe, Germany) in standard cell culture flasks (Greiner Bio-One, Frickenhausen, Germany). Prior to transfection, cells were plated onto 48-well culture plates (Greiner) or onto glass cover slips (placed in 24-well plates) coated with Collagen A (Merck Millipore, Darmstadt, Germany). Cells were transfected with the indicated cDNA expression vector constructs (300ng DNA per transfection in 48-well format) using X-tremeGENE HP reagent (Roche, Mannheim, Germany). A non-relevant plasmid was used to equalize DNA amounts where applicable. Cells were harvested or analyzed within 40–48 h after transfection. In this study 11 independent transfection experiments were conducted with various samples in duplicates each and multiple repeats. Prior to cell harvest for Western blotting (whole cell lysates, transfection sample I) and EMSA (nuclear extracts, duplicate transfection sample II), EGFP fluorescence intensities were measured in 7 out of the 11 transfection experiments by automated microscopy in live cells using a FluoroSpot Analyzer (CTL Immunospot, Bonn, Germany) which also confirmed equal cell numbers, equal transfection efficacies and uniform plating (2–3 scans each). Fluorescence values were quantified with ImageJ. In addition, intensities and subcellular distributions of EGFP signals were determined by conventional epifluorescence microscopy in 10 of the 11 transfection experiments using a Zeiss Axio Observer with a 20x objective and at least two images of each transfection sample were assessed using the Zeiss Zen software. In co-transfections involving GOF-NIK, p100-to-p52 conversion was confirmed by (partial) relocation of the fluorescence signal from the cytoplasmic compartment to the nucleus in transfected cells. In pre-experiments suitable amounts of GOF-NIK expression vector (10-12.5 ng per transfection in 48-well format) ensuring efficient conversion with only minor effects on cell morphology were determined by titration. In multiple repeats, identical results or only minor differences were observed for the overexpressed wildtype and mutant proteins, respectively.

EBV-transformed lymphoblastoid B-cell lines were generated by treatment of ficoll-isolated PBMCs with Epstein-Barr-Virus (EBV) containing B95-8 cell culture supernatants. Cells were grown in RPMI (Roswell Park Memorial Institute) 1640 medium supplemented with 10% FBS and 1% penicillin/streptomycin. CD40L was obtained from Cell Signaling. The EBV cells used in this study (including five different healthy donor lines, and two independently generated lines from the index patient) were inhomogeneous and showed variable growth kinetics. From six independent CD40L stimulation experiments with variable conditions and multiple cell preparations from unstimulated cells we mostly obtained only inconclusive and ambiguous results which did not allow for consistent conclusions regarding p100/p52 expression and which led us to resort to an in vitro overexpression model.

### Analysis of p100/p52 Expression by Flow Cytometry

Intracellular staining of healthy control and patient-derived EBV-immortalized lymphoblastoid cells was performed using the Cytofix/Perm III Kit (BD Biosciences, Heidelberg, Germany) according to the manufacturers’ instructions. Briefly, cells were incubated with Zombie violet (Biolegend) for 15 min and subsequently fixed by addition of Cytofix at 37°C for 10 min and permeabilized with PermIII on ice for 30 min. Cells were washed twice with PBS/0.5% BSA and stained for p100/p52 (#3017; Cell Signaling; monoclonal rabbit), CD19 BV421 and CD21 PeCy7. The anti-p100/p52 antibody recognizes an N-terminal epitope and cannot distinguish between p100 and p52. Cells were measured on a LSR Fortessa (BD Biosciences).

### Cell Isolation, Cultivation and Activation

PBMCs of were isolated from EDTA blood by Ficoll density centrifugation. B cell activation was performed by cultivation of 0.5 × 10^6^ PBMCs with 10 µg/ml goat-anti-human IgM (F(ab’)2 (Southern Biotech; Biozol, Eching, Germany), 20 ng/ml BAFF (kindly provided by Pascal Schneider, University of Lausanne, Switzerland) or without stimulation. After 16 h cells were washed and stained with the respective antibodies in Brilliant stain buffer (BD Biosciences, Heidelberg, Germany). Samples were measured on a LSR Fortessa (BD Biosciences). Antibodies were: ICOS-L PE (2D3), CD86 (IT2.2) PerCP/Cy5.5, CD21 (Bu32) PE/Cy7, CD83 (HB15e) APC, CD19 (HIB19) APC-Cy7, CD38 (HIT2) BV421, IgD (IA6-2) BV510, CD62L (DREG56) BV711; all from BioLegend (Amsterdam, The Netherlands). CD69 (FN50) FITC and CD27 (L128) BV605 were purchased from BD Biosciences.

### Generation of cDNA Expression Vectors and Site-directed Mutagenesis

The cDNAs encoding the human p100 (isoform 1; NM_001077494.3), and a constitutively active variant of human NF-κB-inducing kinase (ΔN325; GOF-NIK) lacking the N-terminal 325 amino acids of NIK [38], were cloned by RT-PCR from an in-house healthy donor RNA source. Restriction enzyme sites for cloning purposes were introduced with the PCR primers. The cDNA encoding the wildtype p52 was cloned from the p100 cDNA with the reverse primer introducing a stop codon after amino acid residue 447. N-terminally EGFP-tagged wildtype p100 and p52 were generated by introducing the cDNAs into the pEGFP-C1 vector (Clontech, Takara, Saint-Germain-en-Laye, France). Sequence variants were introduced into the cDNAs by site-directed mutagenesis using overlap-extension PCR. The cDNAs encoding the shorter 415 and 405 amino acids p52 forms were cloned from their longer parental versions using suitable reverse primers to introduce stop codons. All cDNA constructs were verified by Sanger sequencing.

### Western Blot Analyses

Cells were washed with phosphate-buffered saline (PBS) and lysed in radio-immunoprecipitation assay (RIPA) buffer (50 mM Tris pH 8, 1% Igepal, 0.5% sodium-deoxycholate, 150 mM NaCl, 1 mM ethylenediaminetetraacetic acid (EDTA), 0.1% sodium dodecyl-sulfate (SDS) supplemented with fresh protease inhibitors. Supernatants were subjected to discontinuous 5%/9% Bis-Tris polyacrylamide gel electrophoresis or standard SDS-PAGE and blotted onto PVDF membranes (Merck Millipore) using standard procedures. Primary antibodies were: rabbit anti-NF-κB2 p100/p52 #4882 and rabbit anti-Phospho-NF-κB2 p100 (Ser866/870) #4810; (both from Cell Signaling, Frankfurt, Germany); mouse-anti-GAPDH #G8795 or mouse-anti-beta-Actin #1978 (both from Sigma), rabbit anti-vav1 #SAB4300432 (Merck) and mouse anti-α-tubulin #HRP-66031 (Proteintech, Planegg-Martinsried, Germany). Membranes were processed either for signal detection with an Odyssey infrared scanner (LI-COR Biosciences, Bad Homburg, Germany) using IRDye-coupled goat-anti-rabbit and goat-anti-mouse secondary antibodies (LI-COR) or for chemiluminescence. In this study, samples from 11 transfection experiments (*N* = 11) were analyzed on 14 Western blots (*n* = 14), most in multiple repeats, showing consistent results for overexpressed wildtype and mutant proteins, respectively. EGFP-p52-R261W (*N* = 9; *n* = 12); EGFP-p100-R261W (*N* = 5; *n* = 3) and EGFP-p100-R261W plus GOF-NIK (*N* = 9; *n* = 12).

### Fluorescence Imaging

Cells were seeded onto collagenized glass coverslips (placed in 24-well plates) and transfected with the indicated constructs. Cells were rinsed with PBS, fixed with 4% paraformaldehyde (PFA) and permeabilized with 0.25% Triton-X100. Nuclei were stained with Hoechst 33342 (Sigma/Merck, Darmstadt Germany) and coverslips were mounted onto standard glass slides (Langenbrinck, Emmendingen, Germany). Microscopic images were taken on a Zeiss LSM710 equipped with a 63x/1.40 N.A. oil immersion objective or using a Zeiss Axio Observer equipped with a 40x/0.75 N.A. objective and processed with the Zeiss ZEN software (all from Carl Zeiss, Jena, Germany).

### Electrophoretic Mobility Shift Assay (EMSA)

Nuclear protein extracts were prepared from transfected cells according to a previously published methods [39, 40]. DY681-labelled annealed oligos (forward 5`– AGT TGA GGG GAC TTT CCC AGG C – 3` and reverse 5`- GCC TGG GAA AGT CCC CTC AAC T -3`) were used as the DNA probe. Binding reactions were carried out at room temperature in 1x binding buffer (10mM Tris pH 7.4; 1mM EDTA; 100mM KCl; 0.25mM DTT; 0.25% Tween-20; 5% glycerol; 0.01% BSA; 100ng/µl poly-dI: dC) in a total volume of 10 µl. Samples were separated on 6% polyacrylamide / 1x TAE mini gels and scanned with an Odyssey infrared analyzer (LI-COR Biosciences, Bad Homburg, Germany). The nuclear extract samples from 11 transfection experiments (*N* = 11) were analyzed for DNA-binding activity on a total of 35 EMSA gels (*n* = 35), most in multiple repeats with either identical results or with only minor differences for the overexpressed wildtype and mutant proteins, respectively. EGFP-p52-R261W (*N* = 9; *n* = 28); EGFP-p100-R261W (*N* = 5; *n* = 14) and EGFP-p100-R261W plus GOF-NIK (*N* = 9; *n* = 27).

### Calcium Flux Assay

Frozen PBMCs were preincubated for 30 min at 4 °C with anti-CD4-PE and anti-CD8-APC-Cy7 or with anti-CD19-PE-Cy7 (all from BD Biosciences: #565999; #557834; #557835). Cells were then loaded with 4.5 µM of Indo-1 (ATT Bioquest, #21036) in RPMI medium with 1% FBS and incubated for 45 min at 37 °C in the dark. After three washes with warm PBS, the cell concentration was adjusted to 2 × 10^6^ cells per 500 µl in RPMI medium with 1% FBS. Cells were then stimulated with either 1 µg of anti-CD3-Biotin or 0.5 µg of anti-IgM-Biotin (both from Biolegend: #317319; #314504) and baseline fluorescence of intracellular Ca^2+^ flux was recorded for 60 s. NeutrAvidin (Thermo Fisher, #31000) was then added and fluorescence was measured for up to 5 min. 1 µM of Ionomycin (Cell Signaling, #9995) was used as a positive control of the experiment. Fluorescence was acquired using an LSR Fortessa flow cytometer (BD Biosciences) with FACS Diva Software. Data analysis was performed using FlowJo version 10.10.

## Results

### Case Report: German Family with Unspecified Antibody Deficiency

The index patient (II-1, female, born in 1985) suffered from upper recurrent tonsillitis and severe sinusitis since childhood, requiring intravenous antibiotic therapy approximately twice per year. At the age of 10, she developed arthritis in the right ankle joint, however serum rheumatoid antibodies were not identified. Hence, this was classified as an episode of seronegative arthritis, which resolved without treatment at age 11. She had no further episodes of autoimmunity. A tonsillectomy was performed at 10.5 years of age, however, this did not lead to an improvement in the upper respiratory tract infection frequency or severity. She did not suffer from recurrent viral or opportunistic infections. At 15 years of age, her IgG level was found to be 5.9 g/l, while her IgA, IgM and IgE levels remained within the normal range. IgG1, IgG2 and IgG3 subclasses were reduced. Specific antibody levels to tetanus, diphtheria, and *Haemophilus influenzae* type B were above the protective threshold. Pneumococcal vaccine responses were not assessed. Due to recurrent upper respiratory infections, immunoglobulin replacement therapy was started at that time. At 28 years of age, the immune phenotype of the patient was largely normal, with normal proportions of T- and B- cells and very slightly reduced NK cells. CD4 + and CD8 + T cell subsets, including regulatory T cells (Treg) and circulating T follicular helper cells were unremarkable but effector memory CD8 + T cells were reduced. B cell subpopulations showed reduced unswitched and switched memory B cells (Table 1). Two years later, her IgM dropped below the normal reference range (0.25 g/L). Her CRP remained persistently elevated at regular follow-up intervals between 10 and 40 ng/l, without clinical symptoms of infection or inflammation. A CT Thorax performed at 38 years of age showed no signs of bronchiectasis and was otherwise unremarkable.

**Table 1:** Laboratory features of subject I-1 (father of index patient) and patient II-1 (index patient)

Test	I-1 (65 years)	II-1 (28 years)	II-1 (30 years)	II-1 (39 years)	Reference values for I1 / II1
IgG [g/l]	7.74*	11.4*	6.73* (↓**)**	8.54*	7.00–16.00
IgA [g/l]	1.8	0.88	0.73	0.95	0.70–4.00
IgM [g/l]	0.59	0.41	**0.25 (**↓**)**	**0.29 (**↓**)**	0.40–2.30
Leucocytes [/µl]	6620	6710	9270	6120	3900–9800 / 4000–10,400
Lymphocytes [/µl]	2138	1590	1574	1720	1000–2800
lymphocytes [%]	32.30	**23.70 (**↓**)**	**17.00 (**↓**)**	28.10	27.00–34.00
CD3 + T cells [/µl]	1614	1312	1352	1410	700–2100
CD3 + T cells [%]	75.5	82.50	**85.90 (**↑**)**	82.00	55.00–83.00
CD3 + CD4+ T cells [/µl]	862	899	946	1063	300–1400
CD3 + CD4+ T cells [%]	40.30	56.50	**60.10 (**↑**)**	**61.80 (**↑**)**	28.00–57.00
CD3 + CD8+ T cells [/µl]	736	401	389	343	200–900
CD3 + CD8+ T cells [%]	34.4	25.20	24.70	19.90	10.00–39.00
CD4/CD8 ratio	1.17	2.24	2.43	3.10	1.00–3.60
TCR gamma/delta + in CD3 + T cells [%]	1.53	2.40			1.00–12.00
TCR alpha/beta + in CD3 + T cells [%]	97.80	97.10			88.00–98.00
CD4-CD8- in TCR alpha/beta + T cells (DNT) [%]	0.81	0.70			0.40–2.50
CD19 + B cells [/µl]	272	184	125	186	100–500
CD19 + B cells [%]	12.7	11.60	7.90	10.80	6.00–19.00
CD56 + CD16+ NK cells [/µl]	248	**89 (**↓**)**	95	122	90–600
CD56 + CD16+ NK cells [%]	11.6	**5.60 (**↓**)**	**6.10 (**↓**)**	7.11	7.00–31.00
DR + in CD3 + T cells [%]	9.41	**1.90 (**↓**)**			4.00–18.00
DR + in CD3 + CD4+ T cells [%]	6.13	**1.80 (**↓**)**			3.00–12.00
CD45RA + in CD3 + CD4+ T cells [%]	18.4	47.20			13.00–56.00 / 21.00–58.00
CD45RO + in CD3 + CD4+ T cells [%]	74.2	48.10			35.00–82.00 / 35.00–73.00
DR + in CD3 + CD8+ T cells [%]	12.9	**2.10 (**↓**)**			4.00–32.00
Naive CD8 + T cells (CD28 + CD27+ CD45RA + in CD3 + CD8+ T cells) [%]	12.00	**73.60 (**↑**)**			7.00–58.00 / 23.00–73.00
Memory CD8 + T cells (CD28 + CD27+ CD45RA- in CD3 + CD8+ T cells) [%]	**8.21 (**↓**)**	**12.10 (**↓**)**			21.00–57.00/ 13.00–43.00
Early CD8 + effector cells (CD28-CD27 + in CD3 + CD8+ T cells) [%]	**1.84 (**↓**)**	7.30			3.40–17.00
Late CD8 + effector cells (CD28-CD27- in CD3 + CD8+ T cells) [%]	**61.1 (**↑**)**	7.30			1.10–49.00 / 1.60–36.00
CD19 + B cells [% of lymphocytes]	13.4	7.20			4.80–22.70 / 4.70–18.20
IgM + IgD+ B cells [%]		**96.35 (**↑**)**			67.30–91.80
IgM-IgD- B cells [%]		**3.11 (**↓**)**			7.70–32.00
IgD+CD27- naive B cells [%]	83.4	**89.47 (**↑**)**			36.4–84.00 / 43.20–82.40
IgD+CD27 + B cells [%]	6.70	**6.88 (**↓**)**			3.10–20.80 / 7.20–30.80
IgD-CD27+ (class-switched memory) B cells [%]	**4.88 (**↓**)**	**2.76 (**↓**)**			5.10–31.80 / 6.50–29.20
IgA + B cells [%]	**2.07 (**↓**)**	**1.72 (**↓**)**			2.50–10.90 / 2.70–14.80
IgG + B cells [%]	2.50	**0.97 (**↓**)**			2.50–14.80 / 3.80–13.60
CD21-CD38- B cells [%]	2.71	0.83			1.00–6.30 / 0.80–7.70
Transitional B cells [%]	0.88	2.05			0.80–4.40 / 0.60–3.50
Plasmablasts [%]	**0.15 (**↓**)**	0.80			0.20–2.80 / 0.40–3.60

Numbers in bold with arrows indicate values above (↑) or below (↓) the normal range compared with age-matched controls. Ranges are listed on the right based on [41–43]. *: under IgG replacement

The patient is currently 40 years old and does not suffer from recurrent infections under immunoglobulin replacement therapy. Since commencing immunoglobulin replacement therapy, her IgG trough level remained within protective levels between 8 and 10 g/l, except during a short episode of a treatment administration lapse at 30 years of age (Table 1). At regular clinical follow-up intervals, the patient denied symptoms of endocrine dysfunction, such as fatigue, unexplained weight changes, dizziness or hypotension. Therefore, no endocrine laboratory screening was performed.

**Fig. 1:**
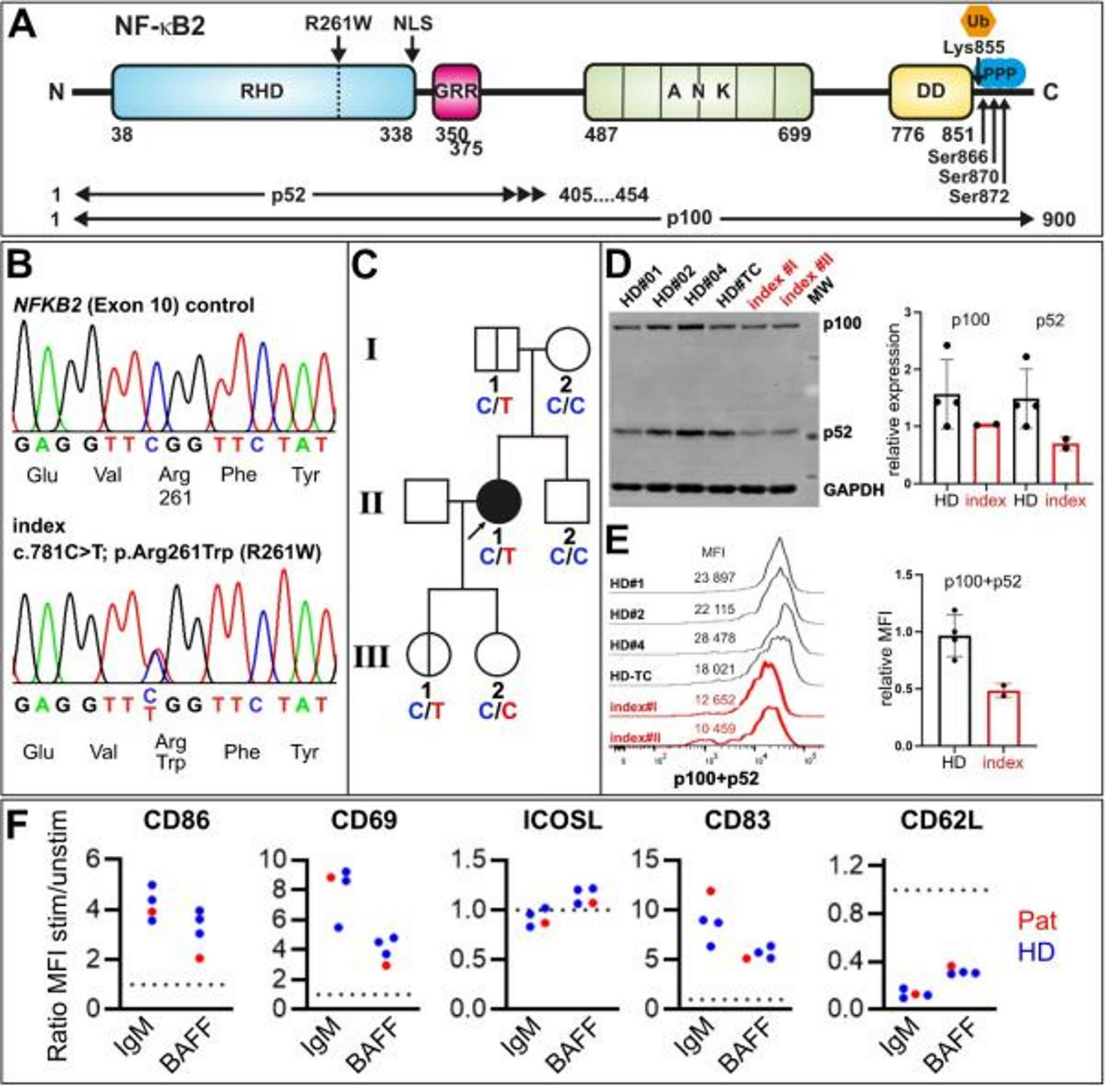
Segregation of the heterozygous *NFKB2* missense variant c.781C>T in a German family with hypogammaglobulinemia is associated with reduced p52 levels and impaired NF-κB2 signaling. **A** Domain structure of p100/p52 and localization of the missense variant p.Arg261Trp (R261W) within the C-terminal part of the Rel-homology domain. Protein domains of the p100 precursor (long horizontal double arrow) comprise the N-terminal Rel-homology domain (RHD), the nuclear localization sequence (NLS), the central glycine-rich region (GRR), and the C-terminal Ankyrin-repeat domain (ANK) and the death domain (DD). Pathway stimulation leads to NIK-dependent phosphorylation of two of the three Serin residues (S866 and S870) near the C-terminus and subsequent ubiquitination at K855. The mature p52 form (short horizontal double arrow), originates from limited proteasomal processing of the C-terminal half of p100. The ‘cleavage site’ which generates the C-terminal end of p52 is reported differently in the literature and is indicated by triple arrow heads. Amino acid positions are indicated by numbers. **B** Sanger sequencing confirms the heterozygous c.781C>T variant, predicting a single amino acid change p.Arg261Trp (R261W) in the index patient. **C** The pedigree of the affected family. Family members tested by Sanger sequencing are indicated. Circles, female; squares, male; filled black symbol, affected individual, divided symbol, unaffected carrier; open symbol, healthy member. The arrow points toward the index. **D** Western blot of whole cell lysates derived from EBV-transformed B lymphocytes shows p100 levels in the lower range and strongly reduced p52 in two independently generated lines from the index patient compared to three healthy donors. Relative normalized densitometric values are shown on the right. **E** Reduced overall NF-κB2 expression (p100 plus p52) in EBV-B cell lines derived from the index patient (red) compared to four healthy donor controls (black). Mean fluorescence intensity (MFI) values are indicated. The relative MFI are shown on the right. Identical cell preparations were used in D and E. **F** Ratio of the mean fluorescence intensities (MFI) stimulated and unstimulated for CD86, CD69, ICOS-L, CD83 and CD62L gated on naïve IgDpositive-CD27negative B cells after stimulation with BAFF or anti-IgM in three healthy donor controls (HD, blue) and the patient (red). In patient cells, CD86 and CD69 are slightly less increased after BAFF stimulation. The gating strategy and representative histograms are shown in Supplementary Figure S3

To test whether a genetic defect accounts for the disease phenotype, the index patient (II-1) underwent routine genetic testing by Next Generation Sequencing (NGS) as previously described [36, 37]. Whole exome sequencing (WES) revealed a heterozygous missense variant c.781C>T in *NFKB2*, predicting the substitution of one amino acid p.Arg261Trp (R261W) within the Rel-homology domain of p100/p52 (Fig. 1A). Most of the known pathogenic *NFKB2* variants affect the C-terminal phospho-degron domain of p100, rendering the precursor non-processable and are, amongst other symptoms, associated with early-onset primary immunodeficiency, antibody deficiency and profoundly impaired B-cell differentiation and frequent T-cell mediated autoimmunity as well as endocrinological abnormalities [4, 7–11]. Compared to published *NFKB2* cases, disease expression in our index patient was relatively milder suggesting a rather ‘non-typical’ p100/p52 defect (if any), consistent with the affected amino acid position far from the C-terminal end. Subsequent Sanger sequencing (Fig. 1B-C) showed that she inherited the *NFKB2* variant from her father (I-1; born in 1959), who was clinically asymptomatic. The father’s laboratory assessment showed marginally reduced IgG levels, IgA and IgM levels within reference range and an impaired polysaccharide pneumococcal vaccine response. His immunological phenotyping showed a reduction in memory and early effector CD8⁺ T cells, along with an increase in late effector CD8⁺ T cells (Table 1). Memory B cells were decreased, with a more pronounced reduction in IgA⁺ B cells, as well as a decrease in plasmablasts. He was commenced on immunoglobulin replacement therapy as a prophylactic measure. The elder of the two daughters (III-1 born in 2011) of the index patient carries the same *NFKB2* variant and has so far remained clinically unaffected. No detailed immunological phenotyping was performed. The younger daughter (III-2 born in 2015), her mother and her brother are wildtype for *NFKB2* (Fig. 1C) as confirmed by Sanger sequencing.

The index patient has previously been reported (alias: patient-171) by us to carry a copy number variant (CNV) of uncertain significance in the putative promoter region of *TRIM22* (tripartite motif containing 22), comprising a 9.3 kb duplication of the first four exons, in addition to the *NFKB2* missense variant [36]. *TRIM22* variants have been associated with inflammatory bowel disease [44], yet TRIM22 is not known to be involved in B cell development or antibody deficiencies.

In addition, the index patient is compound heterozygous *in trans* [c.439G>A];[c.2391C>A] for two missense variants in exons 4 and 26 of *VAV1* (vav guanine nucleotide exchange factor 1) both causing single amino acid changes [p.Asp147Asn];[p.Asp797Glu] ([D147N];[D797E]. She inherited the D147N allele from her father (I-1) which was inherited by her younger daughter (III-2), while the older daughter (III-1) inherited the D797E allele. D147N was listed in gnomAD (allele count: 5) and ClinVar (one carrier) and had moderate variant effect predictions (SIFT: deleterious; PolyPhen: possibly/probably damaging; CADD: 28.8; REVEL: 0.29.). D797E was not reported in population databases (SIFT deleterious; PolyPhen possibly damaging; CADD: 22; REVEL 0.186). Since haploinsufficiency of VAV-1 has previously been associated with T-cell driven CVID and mediates intracellular calcium release and activation of transcription factors such as NFAT and NF-κB in T and B cells, respectively [45, 46] we tested both, VAV1 expression and calcium flux in patient-derived PBMCs (Supplementary Figure S1). While Western blotting indicated reduced VAV1 levels in the index patient and her father, calcium release in stimulated B and T cells was within the normal range. We therefore do not consider the identified *VAV-1* missense variants as pathogenic although we cannot exclude a modifying effect.

### Identification of a Detrimental *NFKB2* Missense Variant as the Genetic Cause of Hypogammaglobulinemia

The heterozygous *NFKB2* missense variant (c.781C>T; p.Arg261Trp; R261W) within the Rel-homology domain affects both, the p100 precursor protein and its processing product, the mature p52 transcription factor subunit (Fig. 1A). The sequence change (dbSNP ID: rs769218279) is listed three times in gnomAD (https://gnomad.broadinstitute.org; dataset v.4.1.0; Variant ID: 10-102398226-C-T), and twice in ClinVar (www.ncbi.nlm.nih.gov/clinvar; Variation ID 546654) with uncertain significance (accessed April 23, 2026). One of the entries represents the current case. ACMG criteria were: PM1, PM2, PP3, (https://wintervar.wglab.org/). Variant effect predictions were: SIFT deleterious; PolyPhen: probably damaging; CADD: 34; REVEL 0.924. R261W has been shown to affect the surface of the dimerization domain of p52 [47] and has been identified as a somatic sequence variant in endometrial, kidney and lung carcinoma tissue samples (https://cancer.sanger.ac.uk/cosmic; Genomic Mutation ID COSV50045881).

A sequence alignment indicated Arg261 of the non-canonical p100/p52 to correspond to Arg284 of the canonical p105/p50, a residue known to be affected by the detrimental missense change R284P [33]. We therefore hypothesized that the single amino acid change R261W in *NFKB2* might cause a similar defect with protein loss. Western blot analysis of unstimulated EBV-immortalized lymphoblastoid cells derived from the index patient showed moderately diminished levels of p100 (72.6 ± 21.4% of HD levels), while p52 was decreased to approximately half (51.0 ± 14.9% of HD levels) compared to healthy donor controls (Fig. 1D). Similarly, in CD40L-stimulated EBV-B cells, we found reduced p52 levels, while p100 and phospho-p100 were less affected (Supplementary Figure S2). However, various of our attempts to determine p100 and phospho-p100 levels by Western blotting mostly remained inconclusive, likely due to the variable expression in our cell lines, while reduced p52 was repeatedly examined in patient-derived cells (data not shown). When we used flow-cytometric analysis in patient-derived EBV lines, we observed the expression of NF-κB2 (total levels of p100 plus p52) to be reduced to 37–70% of the levels in healthy donor cells (Fig. 1E). Since Western blot analyses suggested that p52 is likely to account for a larger proportion of the protein loss than p100, we suspected that R261W might destabilize p100 levels, but primarily affects p52 in a destructive way, probably leaving very few (or no) mutated proteins which causes a shortage of the mature transcription factor subunits.

To address the functional capacity of B cells carrying the *NFKB2* R261W variant in vitro, PBMCs were stimulated with BAFF to activate the alternative NF-κB2 pathway or with anti-IgM to selectively stimulate signaling *via* the canonical NF-κB1 pathway (Fig. 1F; Supplementary Fig. 3). Flow cytometry indicated that BAFF-dependent upregulation of CD86 and to lower extend of CD69 was reduced on naïve B cells derived from the patient compared to the healthy donor controls. However, both markers were normally induced after anti-IgM stimulation. The upregulation of ICOSL and CD83 as well as the downregulation of CD62L was not remarkably different from healthy controls. These findings confirm functional alterations in alternative NF-κB2 signaling while BCR-mediated signaling was normal. However, tests for reduced p100/p52 expression in primary cells did not yield clear results (data not shown).

### The R261W Missense Variant Mitigates the Sustainability of the NF-κB2 Transcription Factor Precursor p100 and Causes Subnuclear Mis-localization of the Mature p52 Upon Overexpression

**Fig. 2:**
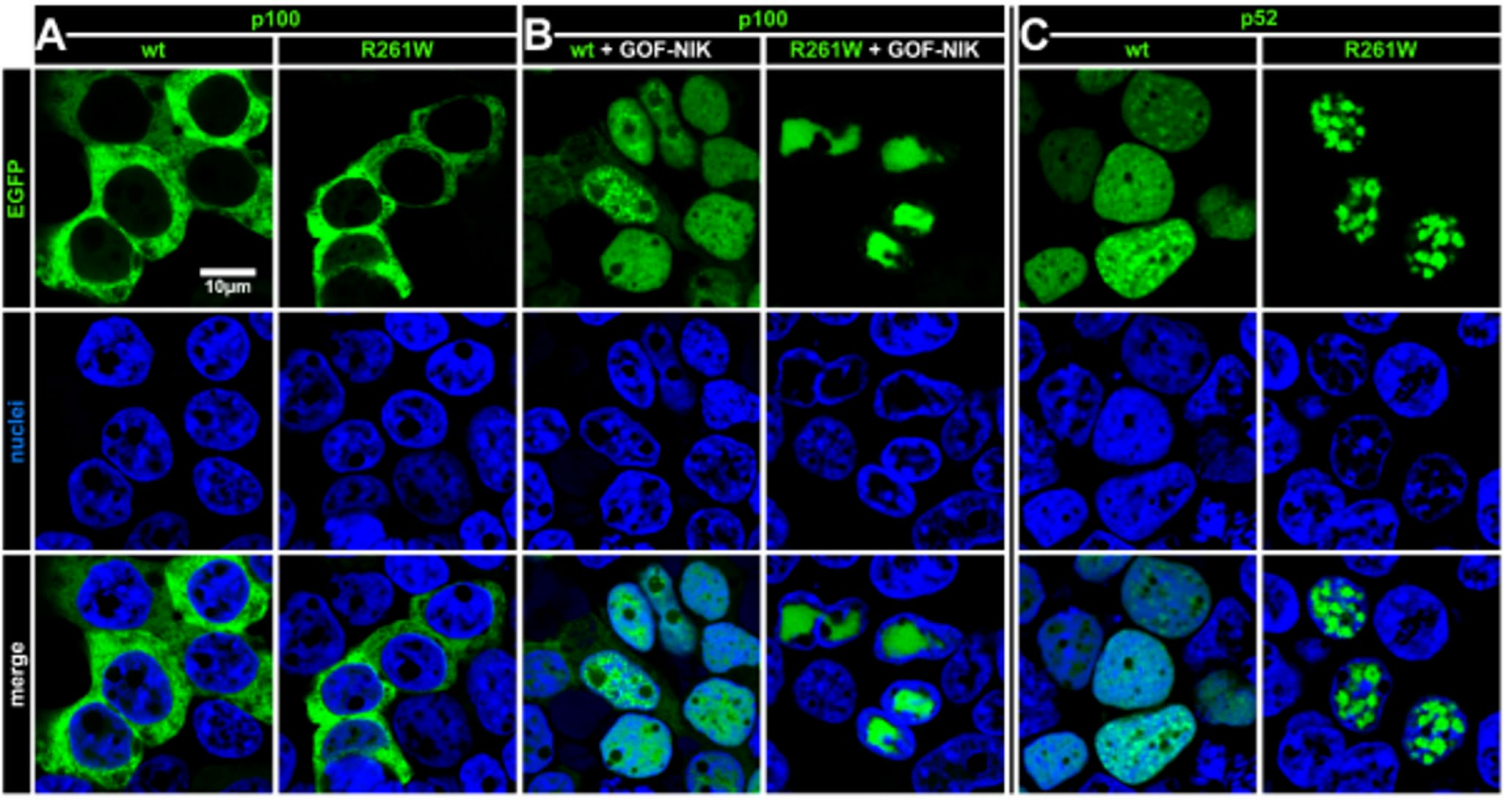
The NF-κB2 missense variant R261W causes sub-nuclear mis-localization and aggregation of p52. Confocal fluorescence microscopy of HEK293T cells transiently transfected with expression vectors (300ng) encoding EGFP-tagged wildtype or R261W-mutant p100 or p52 as indicated (green). A non-processable truncated variant R853X and an empty EGFP-vector control were included as shown in Supplementary Figure S4. DIC-overlay images are shown in Supplementary Figure S5. Nuclei were stained with Hoechst33342 (blue). A scale bar is indicated in the upper left panel. Paradigmatic images are shown representing the most prominent aspects. **A** Both, p100-wt and p100-R261W localize exclusively to the cytoplasm. Please note: p100-R261W typically gains weaker intensities compared to the wildtype control (not shown), suggestive of lowered stability. Fluorescence intensities were adjusted for best visibility and do not reflect the relative expression levels (please see low-resolution images in Supplementary Figure S4). **B** Co-expression of wildtype or R261W-mutant p100 together with a constitutively active variant of the NF-κB-inducing kinase (GOF-NIK) to enforce p100-to-p52 processing. Upon GOF-NIK-mediated processing of its cytoplasmic EGFP-p100 precursor, the endogenously generated EGFP-p52-wt localizes with a homogeneous distribution within the nuclei. In contrast, the endogenously generated EGFP-p52-R261W mutant accumulates in dense aggregates in the centers of the nuclei. 3D-reconstructed Z-scans are shown in Supplementary Figure S6. **C** The artificially expressed nuclear wildtype EGFP-p52 (short-cutting the generation of nuclear p52 from its cytoplasmic p100 precursor), like its endogenously generated counterpart, shows a homogeneous subnuclear localization, while the directly expressed EGFP-p52-R261W mutant proteins again accumulate within dense structures inside the nuclei. Please note the unique subnuclear localization patterns of the endogenously generated and the ectopic EGFP-p52-R261W. Please also see the 3D-images in Supplementary Figure S6

Because we obtained ambiguous results when analyzing p100 and p52 protein content in EBV cells and primary patient-derived cells, we sought for a better suited model to investigate the effects of the R261W variant on protein stability and function. Therefore, to confirm that R261W causes a severe protein damage, we followed previously introduced and approved in vitro protocols, enabling the functional characterization of mutant NF-κB proteins [33, 34]. The assay employs overexpression of EGFP-fusion wildtype and mutant NF-κB proteins in a routine cell culture model while any observed deviation from the functional properties of the wildtype control indicates a pathogenic protein defect and supports the diagnosis of a NF-κB-related disease. We first generated cDNA expression constructs for N-terminally EGFP-tagged derivates of wildtype p100 (the cytoplasmic precursor) and p52 (the nuclear transcription factor subunit) and subsequently introduced the missense variant *via* site-directed mutagenesis to obtain EGFP-p100-R261W and EGFP-p52-R261W. We then assessed the subcellular localization of the overexpressed proteins in HEK293T cells by fluorescence microscopy. Both EGFP-p100wt and EGFP-p100-R261W were confined to the cytoplasmic compartment in all transfected cells (Fig. 2A), with the mutant protein gaining markedly weaker intensities (Supplementary Figure S4), which suggests reduced protein sustainability.

To promote p100-to-p52 processing, we adapted a firmly-established and elegant experimental maneuver [26], and co-transfected trace amounts of a vector encoding a constitutively active variant of the NF-κB-inducing kinase [38], denominated here as GOF-NIK, together with the vectors encoding the wildtype or mutant p100 precursors (Fig. 2B and Supplementary Figures S4 and S5). Upon overexpression of the EGFP-fused wildtype p100, the co-expressed GOF-NIK caused a relocation of the fluorescence signal from the cytoplasm to the nucleus in a high proportion of the transfected cells, indicating efficient, GOF-NIK-dependent conversion of cytoplasmic p100 precursor proteins into mature nuclear p52 subunits (please see the sections below). While the nuclear fluorescence of the wildtype protein was uniformly distributed, the R261W-mutant p52 proteins, generated from their mutant precursors by the ectopic GOF-NIK activity, accumulated in dense aggregates in the centers of the nuclei (please see the following two paragraphs for details), thereby displacing the genomic DNA to the periphery of the nuclei (Fig. 2B and Supplementary Figures S4 and S5). The cytoplasmic localization and expression level of the C-terminally truncated, non-processable variant p.Arg853* (R853X), which was included as a representative control of the most common types of pathogenic *NFKB2* variants, was indistinguishable from the wildtype control while the EGFP alone had no specific subcellular localization. Both proteins largely remained unaffected when GOF-NIK was co-expressed (Supplementary Figure S4) further confirming the high specificity of the assay.

Upon immediate expression of the EGFP-fused wildtype p52 (to by-pass the NIK-mediated processes which generate p52 from its precursor), the homogeneously distributed fluorescence signals were exclusively detected within the nuclei of all transfected cells (Fig. 2C; Supplementary Figures S5 and S6). In contrast, the missense mutant EGFP-p52-R261W accumulated in high intense spot-like sub-nuclear structures in all cells gaining high overexpression levels, again occupying the centers of the nuclei, and caused morphological aberrations of the nuclei.

The altered expression and localization of the R261W-mutant NF-κB2 proteins resembles the typical pattern previously observed with protein-decaying pathogenic *NFKB1* variants [2, 33, 34, 48]. Formation of characteristic subnuclear aggregates upon overexpression in vitro has previously been established as the most-reliable indicator of protein-damaging missense variants and certain types of truncating variants in *NFKB1* and is hitherto a main diagnostic criterion for pathogenicity. The dynamically accumulating fluorescence signals can result in huge subnuclear structures and is typically seen in cells with high over-expression levels of severely damaged p50 and, as we demonstrate here, of p52 as well. We therefore hypothesized that the R261W variant in *NFKB2* similarly causes intensified protein loss of p100 but particularly of p52.

### The R261W Missense Variant Prevents Durable p52 Expression

**Fig. 3:**
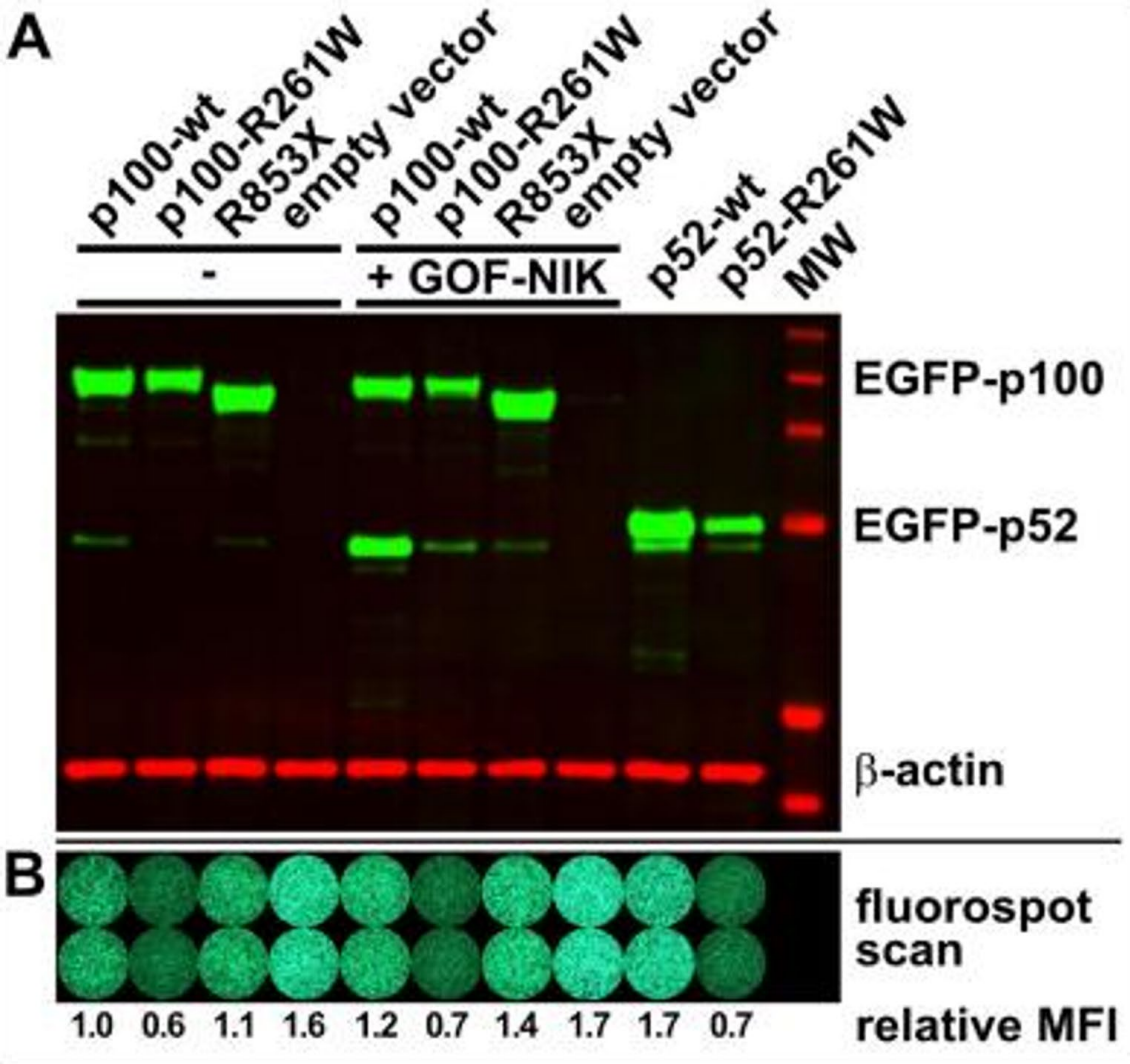
The R261W missense variant causes a deleterious defect with protein loss. HEK293T cells were transiently transfected with expression vector constructs (300ng) either encoding EGFP-fused p100 derivates alone (left) or together with untagged GOF-NIK (12.5ng) to promote p100-to-p52 conversion (middle), or encoding wildtype or mutant p52 (right). The truncated variant R853X and the empty EGFP-vector serve as controls. **A** Proteins were simultaneously detected in whole cell lysates by Western blotting using an antibody directed against an N-terminal epitope of p100 and p52 (both green). An anti-β-actin antibody was used as loading control (red). Representative results of multiple experimental repeats as indicated in Methods are shown. (left) In transfected cells, a small portion of wildtype (and to a much lesser extent of the truncated R853X) but not of the R261W-mutant EGFP-p100 is constitutively converted to EGFP-p52 by endogenous precursor processing. EGFP-p100-R261W typically gained weakened expression levels and could not be elevated using saturating DNA amounts (Supplementary Figure S7). (middle) Co-expression of GOF-NIK strongly promoted processing of the wildtype EGFP-p100 whereas the mutant precursor forms either are only weakly processed or release only unsustainable p52 (R261W) or largely remain unprocessed (R853X). (right) The limited expression level gained with the EGFP-p52-R261W expression construct indicates the deleterious character of the missense variant. Please note: the p52 proteins (still retaining their N-terminal EGFP-tag) generated by endogenous mechanisms are smaller in size than the EGFP-fused 447 amino acid p52 used for ectopic expression (which is likewise further processed into a smaller form). Comparable results were obtained with shorter p52 versions (415 and 405 amino acids; Supplementary Figures S4 and S8) verifying that the mis-localization and decay is due to the R261W missense change but not the extension at the C-terminal end. **B** Automated microscopic scan of the duplicate transfection samples in 48-well format prior to cell harvest to obtain whole cell lysates for the Western blot shown above or nuclear extracts for EMSAs as shown in Fig. 4 and relative fluorescence intensities (MFI) normalized to the EGFP-p100wt samples. EGFP-p100-R261W gained (0.73 ± 0.14)-fold and EGFP-p100-R261W plus GOF-NIK (0.55 ± 0.03)-fold MFI values compared to the wildtype counterpart in 4 and 7 independent experiments, respectively. Please note: The magnitude of any observed deviation may vary depending on the experimental conditions and should not be regarded as a ‘threshold’ for pathogenic effects

To further characterize the deleterious effects of the R261W missense change, we analyzed the overexpressed mutant proteins in transfected HEK293T cells by Western blotting (Fig. 3). The EGFP-fused wildtype p100 gained robust protein levels, consistent with our microscopic observations. In contrast, the p100-R261W variant only reached moderate levels (54.9 ± 3.1% of the wildtype protein), compatible with injurious effects, while expression of the truncated R853X variant (which was included as a non-processable control) was comparable to its wildtype counterpart (Fig. 3; left). Constitutively generated EGFP-p52 - produced from the transfected EGFP-fused precursor proteins by cell-intrinsic processes - was observed in moderate amounts upon overexpression of EGFP-tagged wildtype p100 (5.08 ± 1.55% compared to its precursor), but was only weakly present (1.26 ± 1.72% compared to its precursor and only 19.52 ± 9.64% of wildtype levels) or in some cases undetectable with the EGFP-p100-R261W variant (Fig. 3 left and Supplementary Figure S7). Minor amounts of EGFP-p52 were also generated from the overexpressed EGFP-R853X mutant.

Strongly intensified p100-to-p52 processing (with p52 reaching 146.6 ± 19.3% of p100 levels) mediated by co-delivery of GOF-NIK, was only observed with the EGFP-tagged wildtype p100, whereas only marginal amounts of EGFP-p52-R261W (reaching only 14.1 ± 2.4% of its parental protein and only 6.8 ± 2.0% of the wildtype levels) were generated from its mutant p100 precursor (Fig. 3; middle and Supplementary Figure S7). A minor increase (if at all) was detectable with the truncated R853X variant, confirming its resistance against NIK-mediated activation. Compared to the EGFP-fused wildtype p52, immediate expression of the nuclear EGFP-p52-R261W variant gained substantially lower (21.2 ± 5.5% of wildtype) protein levels (Fig. 3; right), indicating a damaging effect, which is compatible with subnuclear disposition as observed in the microscopic analyses above.

To experimentally short-cut the p100-to-p52 conversion by immediate expression of p52, we used an artificial 447 amino acids protein [24, 25], which (most likely due to an extension at the C-terminal end) has a slightly higher molecular weight than the p52 form generated by endogenous processes (Fig. 3). However, except for reduced expression levels, we did not observe any relevant difference (Supplementary Figures S4 and S8) when we repeated our experiments with 415 and 405 amino acids versions of p52 (415aa wildtype: 82.4 ± 15.6%; 405aa wildtype: 82.4 ± 19.1; 415aa mutant: 46.1 ± 9.1%; 405aa mutant: 47.2 ± 12.6% compared to the 447aa versions each), which better match the size of endogenously generated protein [47, 49, 50]. The even longer 454 amino acids variant (Uniprot Q00653) has not been tested.

Our in vitro results, involving CMV-promoter-driven protein over-expression in HEK293T cells, suggest that the missense mutant p100-R261W precursors have reduced sustainability, which limits the generation of mutant and probably harmful p52 transcription factors. The mutant mature p52-R261W subunits, however, are unsustainable and appear to undergo augmented decay and/or subnuclear deposition. Alternatively, if R261W only affects the stability of p52, permanent processing of p100 to supply and to maintain baseline p52 levels, would limit the total amount of p100. Therefore, in either case, a disease-causing mechanism most likely originates from reduced amounts of p100 and particularly p52, rather than from the presence of dysfunctional proteins.

### Cell-intrinsic Processing of p100-R261W Does not Produce Nuclear DNA-binding Activity Although DNA-binding Ability of Ectopically Expressed p52-R261W Remains Preserved

**Fig. 4:**
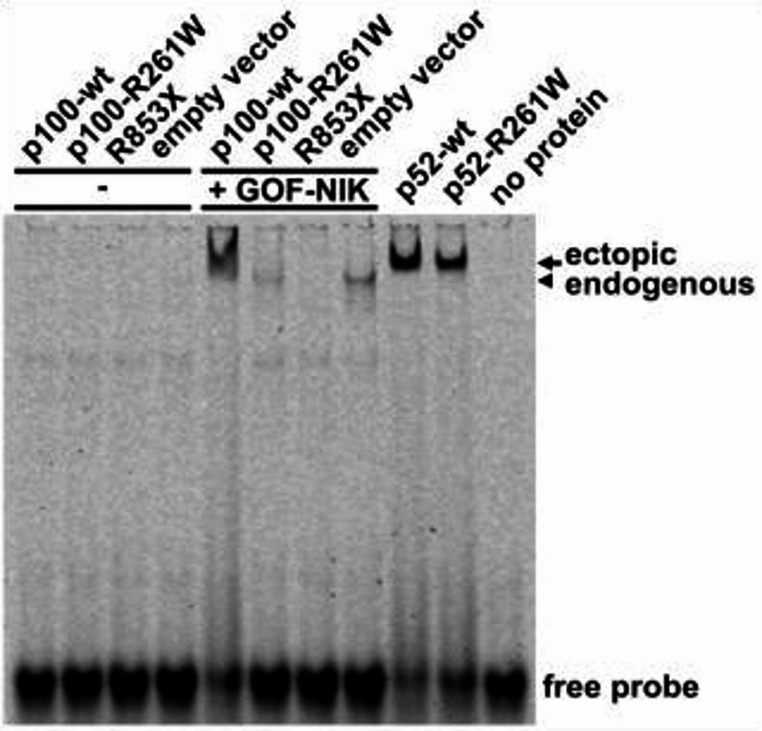
The R261W missense variant abolishes the generation of p52-dependent DNA-binding activities *via* endogenous precursor processing, although DNA-binding itself is retained in ectopically expressed mutant p52. HEK293T cells were transiently transfected with 300ng of the indicated EGFP-fused derivates of p100 or p52 as indicated (and as shown in Fig. 3). NF-κB-specific DNA-binding activities in nuclear protein extracts were analyzed by EMSA. Representative results (please see Methods for details) are shown. (left) In cells transfected with wildtype or mutant EGFP-p100 expression constructs, DNA-binding activity is undetectable, most likely because the processed EGFP-p52 proteins (both wildtype and mutant) are retained within the cytoplasm by excess IκB-activities (presumably mediated by their own overexpressed precursors) or because constitutive processing is low or absent (p100-R261W and R853X). (middle) Co-expression of GOF-NIK (12.5ng DNA) leads to increased p100-to-p52 processing of the wildtype EGFP-p100 (as shown in Fig. 3) and consequently to pronounced nuclear DNA-binding activity, also including endogenous NF-κB proteins. No DNA-binding activity is generated from the EGFP-fused p100-R261W-mutant precursor, even with saturating DNA amounts (please see Supplementary Figure S7). Please note: the smaller sized EMSA bands in EGFP-p100-R261W and the empty vector lanes, most likely correspond to endogenous NF-κB proteins (and are restrained in the cytoplasm by R853X). (right) Upon artificial expression of mature nuclear EGFP-p52 (i.e. not generated *via* processing of cytoplasmic precursor proteins), strong DNA-binding activities are detectable with both, the wildtype and the R261W-mutant. Comparable results were obtained with three differently-sized (447, 415 and 405 amino acids) ectopically expressed p52 variants (Supplementary Figure S8). Under non-saturated conditions, the magnitude of DNA-binding depends on the overexpression levels and protein input

We then tested whether the missense variant R261W prevents the generation of p52-dependent DNA-binding activities (which involves dimerization and nuclear translocation) when p52 is generated *via* intracellular processing of overexpressed mutant p100 precursor proteins. This could, if binding partners from endogenous pools are available, allow the assembly of any hetero- or homo-dimeric NF-κB transcription factor species. In addition, we investigated whether the amino acid change itself interferes with the DNA binding ability of p52. When overexpressed, nuclear p52 is constitutively present in excess and, if dimerization is enabled, the assembly of homo-dimers is likely favored due to stoichiometry. Using electrophoretic mobility shift assays (EMSA) no DNA-binding activity was detectable in nuclear protein extracts when either wildtype or R261W-mutant EGFP-fused p100 alone was overexpressed in HEK293T cells (Fig. 4 left), indicating that constitutive precursor processing is too low or absent (as determined by Western blotting; please see Fig. 3) and/or that nuclear translocation of newly generated p52 is prevented by excess cytoplasmic IκB-activity (most likely mediated by their own parental p100 proteins). No DNA-binding was observed with the truncated R853X variant.

When we co-expressed EGFP-p100 together with GOF-NIK to enhance precursor processing and EGFP-p52 generation, strong DNA binding activity was detectable, (gaining 96.5 ± 6.4% of the levels of the ectopically expressed EGFP-p52; lane 9) only in the wildtype control, but not with the EGFP-fused R261W-mutant precursors or the non-processable truncated variant (Fig. 4 middle). However, GOF-NIK expression was sufficient to increase the nuclear DNA-binding activity of cell-intrinsic NF-κB proteins, discernable by the smaller size of the shifted band. Interestingly, the truncated EGFP-R853X variant, which gains expression levels comparable to its wildtype counterpart, was able to completely block nuclear translocation of endogenous NF-κB proteins, while the low expressed EGFP-p100-R261W mutant was not.

On the other hand, robust DNA-binding activity was obtained upon direct overexpression of both, the EGFP-fused nuclear wildtype p52 and the mutant p52-R261W variant (gaining 61.4 ± 15.6% of wildtype levels), suggesting that the single amino acid change *per se* does not abolish the ability of the mutant transcription factor to bind to DNA (Fig. 4, right).

Together, these observations suggest a scenario in which R261W-mutant precursor proteins can be expressed, but are unsustainable, and are either not further processed to produce mutant p52 transcription factor subunits, or the processing products are instantly removed or sequestered by a protein quality control mechanism to protect the NF-κB system from dysfunctional signaling components. Both the overall mild phenotypes within the affected family and the low frequency of p52-decaying variants observed in IEI patients are consistent with previous reports [7–11], suggesting the heterozygous loss of p52 – or even of p100 and p52 - being usually associated with haplo-sufficiency (non-pathogenic), rather than insufficiency (pathogenic).

## Discussion

Heterozygous damaging sequence variants in *NFKB1* and *NFKB2* have been recognized as molecular causes for IEI [2, 5]. Yet the pathogenic mechanisms underlying the highly variable disease phenotypes are poorly understood, particularly because both NF-κB signaling pathways are central to numerous aspects of immunoregulation. In addition, the molecular defects in either of the encoded proteins are diverse, and the regulation of NF-κB signaling is highly complex.

In the present study we characterized a heterozygous deleterious *NFKB2* missense variant (R261W), causing a severe defect of p52 with protein loss and reduced sustainability of p100. In contrast to *NFKB1* where the most common type of pathogenic variants causes p105/p50 haploinsufficiency [35], *NFKB2*-associated symptomatic cases with assured haploinsufficiency of p52 or of both, p100 and p52 (i.e. without mutant proteins being present) are apparently rare [7–11]. In a recent report, a severely truncating nonsense variant (W270X), which prematurely terminates expression of p100 and thus causes monoallelic loss-of-expression of both, p100 and p52 (rather than ‘secondary’ protein loss) has been associated with a mild CVID phenotype in a single patient, while two mutation carriers of the same family were unaffected [32]. Similar to the patient reported here, the affected individual showed a reduction of switched memory B cells. In addition, cytotoxic effector memory T cells were reduced in the affected patient and one unaffected mutation carrier [32]. Interestingly, the reduction of switched memory B cells and hypogammaglobulinemia is common to patients with all different types of pathogenic p100/p52 variants [5, 7–11, 31]. In contrast, reduced cTFH cells, which are crucial for memory B cell formation and Tregs were not altered in patients with *NFKB2* haploinsufficiency. The integrity of both CD4 + T cell subsets as well as normal NK cells may account for a milder form of hypogammaglobulinemia and an infection only phenotype in these patients compared to the complicated form of immunodeficiency with early onset associated with genetic defects at the C-terminal end of p100.

Clinically, we recommend a baseline assessment of asymptomatic carriers, including a targeted clinical history, T-, B-, and NK-cell levels, B-cell immunophenotyping, immunoglobulin levels and vaccine responses. Following this, it would be prudent to perform annual screening, including clinical history, immunoglobulin levels, and T-, B-, and NK-cell levels in asymptomatic carriers, as data on the natural history of the disease remain largely unknown.

Particularly because the associated disease phenotypes might be mild, i.e. not requiring treatment, or because the mutation carriers might be clinically asymptomatic (and therefore rather haplo-sufficient), heterozygous protein-decaying *NFKB2* variants might be underdiagnosed and underestimated. Hence, in patients diagnosed with ‘mild’ or ‘infection-only’ CVID, ‘selective IgG deficiency’ or ‘unclassified antibody deficiency’ the (re)analysis for damaging *NFKB2* variants – other than those affecting the C-terminal degron – might be worthwhile, if a genetic cause of the disease is assumed and/or if the causality remains unclear. Interestingly, the index patient and her father did not fully meet ESID CVID criteria [51]. Therefore, we suggest maintaining a low threshold for genetic testing in patients in whom a primary immunodeficiency is suspected, irrespective of whether ESID criteria are fulfilled.

Most of the hitherto described pathogenic *NFKB2* variants cause genetic lesions at the C-terminal end of p100, which prevent p100-to-p52 processing and thus result in excess p100 and simultaneous shortage of the source required for generation of p52 [4, 5, 7–11, 26, 27]. The persistent presence of mutant p100 precursor proteins is obviously more harmful than reduced amounts, as its excess can cause severe disease phenotypes [7–11]. Besides providing a reservoir for p52, the p100 precursor exerts additional IκB-like functions by associating with other NF-κB transcription factors [5, 23], including its own processing product p52. Therefore, p100 represents a connecting link between non-canonical and canonical pathways and thus, both NFKB1- and NFKB2-related diseases might share at least some common molecular mechanisms.

The third, less-frequently occurring type of immune-pathogenic *NFKB2* variants comprises a small group of monoallelic centrally truncating mutations [7–11, 28–31]. These predict to completely bypass the p100 precursor stage and – if expressed at all – lead to immediate translation of p52-like proteins, instead of being generated from longer precursors. Known examples are E418X, with a protein size comparable to wildtype p52, or with C-terminal extensions (e.g. R611X and R653X), retaining parts of the ankyrin repeat domain (Fig. 1A). Since the inhibitory activity mediated by p100 is partially lost, such variants have been suggested to cause an overall gain-of-NF-κB signaling [31]. However, the similar-sized p52-like proteins themselves might be functionally equivalent to their wildtype counterpart (such as the 447, 415 and 405 amino acids forms in our study; Supplementary Figure S8). In cases where premature termination of translation causes accelerated mRNA and/or protein decay, C-terminally extended p52-proteins might be undetectable in patient-derived cells [28, 31] which therefore appear as ‘p100/p52 haplo-(in)sufficient when tested by Western blotting. However, some of these variants might not cause a clinical phenotype [28].

In summary, pathogenic *NFKB2* variants can be assigned into distinct protein defect groups, each causing a unique spectrum of disease symptoms [7–11]. The harmful character of truncating variants affecting either the C-terminal, the central/proximal or the N-terminal domains can be assumed with some accuracy. Yet, the pathogenic relevance of each missense variant requires individual functional testing.

Our report provides one experimental option to identify deleterious p100/p52 protein-defects. We are aware that mildly affected or asymptomatic mutation carriers might be unsettled with a genetic diagnosis saying ‘reduced p52 or likewise p100/p52 due to a harmful missense variant’. Functional screening of larger numbers of *NFKB2* missense variants with uncertain significance, for instance as identified in CVID cohorts, will show whether such an approach is reasonable to confirm or clarify a diagnosis.

## Supplementary Information

Below is the link to the electronic supplementary material.

Supplementary Material 1

## Data Availability

All data supporting the findings of this study are available within the paper and its Supplementary Information.

## Abbreviations

ANK: Ankyrin-repeat domain
CVID: Common variable Immunodeficiency
DD: Death domain
DIC: Differential interference contrast
DMEM: Dulbeccco´s Modified Eagle Medium
EDTA: Ethylenediaminetetraacetic acid
EGFP: Enhanced green fluorescent protein
EMSA: Electrophoretic mobility shift assay
FCS: Fetal calf serum
GOF: Gain of function
IEI: Inborn error of immunity
IKK: IκB kinase
NF-κB: Nuclear factor ‘kappa-light-chain-enhancer’ of activated B-cells
NFKB2: Nuclear factor kappa B subunit 2
NIK: NF-κB inducing kinase
NLS: Nuclear localization sequence
PAD: Primary antibody deficiency
PBMC: Peripheral blood mononuclear cells
PID: Primary immunodeficiency
PVDF: Polyvinylidene fluoride
RHD: Rel-homology domain
RIPA: Radioimmunoprecipitation assay
RPMI: Rosewell Park Memorial Institute
SDS: Sodium-Dodecyl-Sulfate
VUS: Variant of uncertain significance
WES: Whole Exome Sequencing
