# Supplementary material for: A Detrimental NFKB2 Missense Variant is Associated with Hypogammaglobulinemia
Source: J Clin Immunol. 2026 Jul 11;46(1):75. doi: 10.1007/s10875-026-02051-9 (PMC13356095; doi:10.1007/s10875-026-02051-9)
Supplement: Supplementary file 1 — Supplementary Material 1 [file 10875_2026_2051_MOESM1_ESM.pdf]

A detrimental *NFKB2* missense variant is associated with hypogammaglobulinemia

Manfred Fliegauf[1,2,], Laura Gamez-Diaz[1,2,3], Pavla Mrovecova[1,2,], Chiara Milena God[2,4], Valerie Flavia Geiger[2,4], Nadezhda Camacho-Ordonez[1,2], Sara Posadas-Cantera[1,2,5], Cliodhna Murray[1,2], Klaus Warnatz[2,4,6], Baerbel Keller[2,4], and Bodo Grimbacher[1,2,3,4,7,8,§]

1 Institute for Immunodeficiency (IFI), Center for Chronic Immunodeficiency (CCI), Medical Center - University of Freiburg, Faculty of Medicine, University of Freiburg, Freiburg, Germany.

2 Center for Chronic Immunodeficiency (CCI), Medical Center - University of Freiburg, Faculty of Medicine, University of Freiburg, Freiburg, Germany.

3 CIBSS – Centre for Integrative Biological Signalling Studies, Albert-Ludwigs University, Freiburg, Germany.

4 Department of Rheumatology and Clinical Immunology, Medical Center-University of Freiburg, Faculty of Medicine, University of Freiburg, Freiburg, Germany.

5 Institute for Microbiology, Medical Center - University of Freiburg, Faculty of Medicine, University of Freiburg, Freiburg, Germany.

6 Department of Immunology, University Hospital Zurich, Zurich, Switzerland.

7 DZIF – German Center for Infection Research, Satellite Center Freiburg, Germany

8 RESIST – Cluster of Excellence 2155 to Hanover Medical School, Satellite Center Freiburg, Germany.

§ Corresponding Author

Bodo Grimbacher, MD
e-mail: bodo.grimbacher@uniklinik-freiburg.de
Institute for Immunodeficiency
Center for Chronic Immunodeficiency (CCI)
Medical Faculty, University Hospital Freiburg
Breisacherstraße 115
79106 Freiburg/Breisgau
Germany

Supplementary Material

Supplementary Figure S1

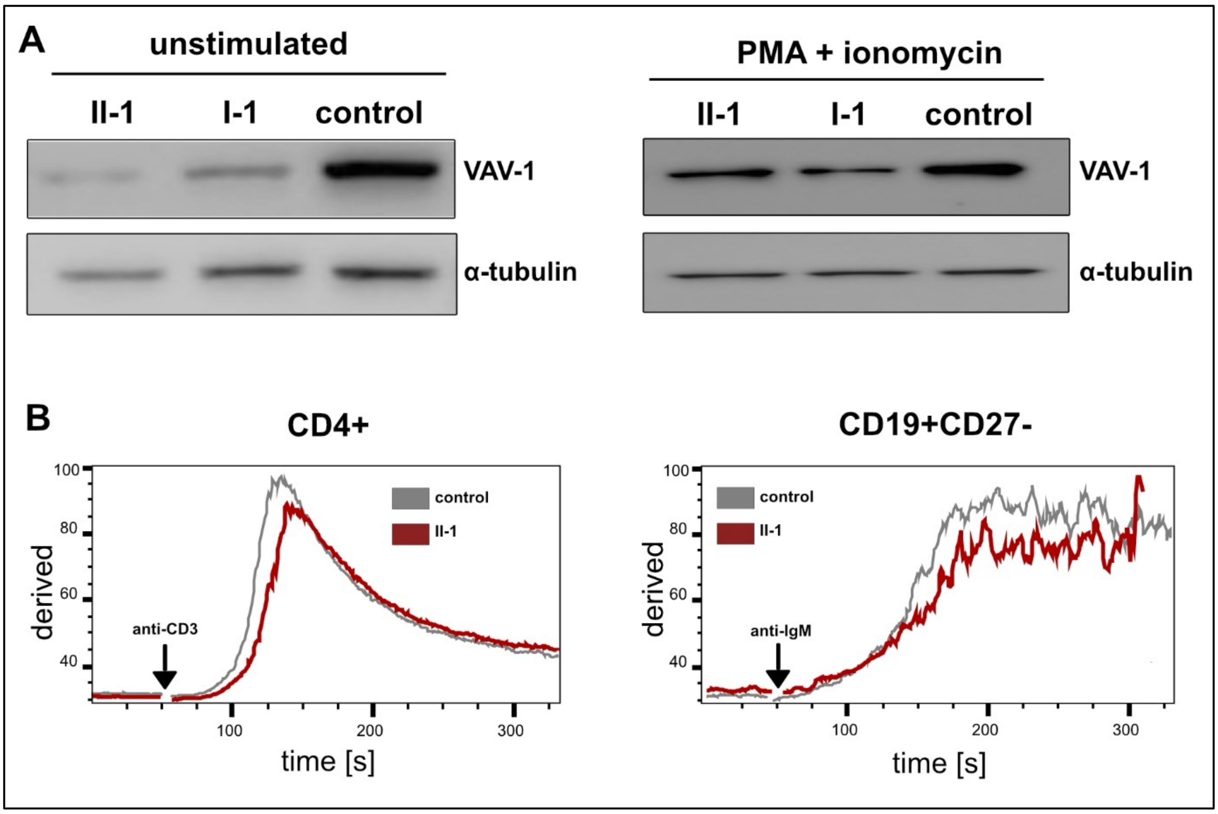

Reduced VAV1 expression and slightly diminished calcium influx in the index patient

(A) Western blot analysis of PBMCs indicates reduced VAV1 levels in the index patient (compound heterozygous *in trans* for [D147N];[D797E]) and her father (carrying the D147N allele only) compared to a healthy control, both before and after stimulation for 4 hours with 20 nM PMA and 500 nM of ionomycin. (B) Calcium release is mildly reduced but within the lower normal range both in CD4+ T cells and in CD19+ B cells from the index patient upon stimulation with 1μg anti-CD3 or 0.5 μM anti-IgM, respectively.

Supplementary Figure S2

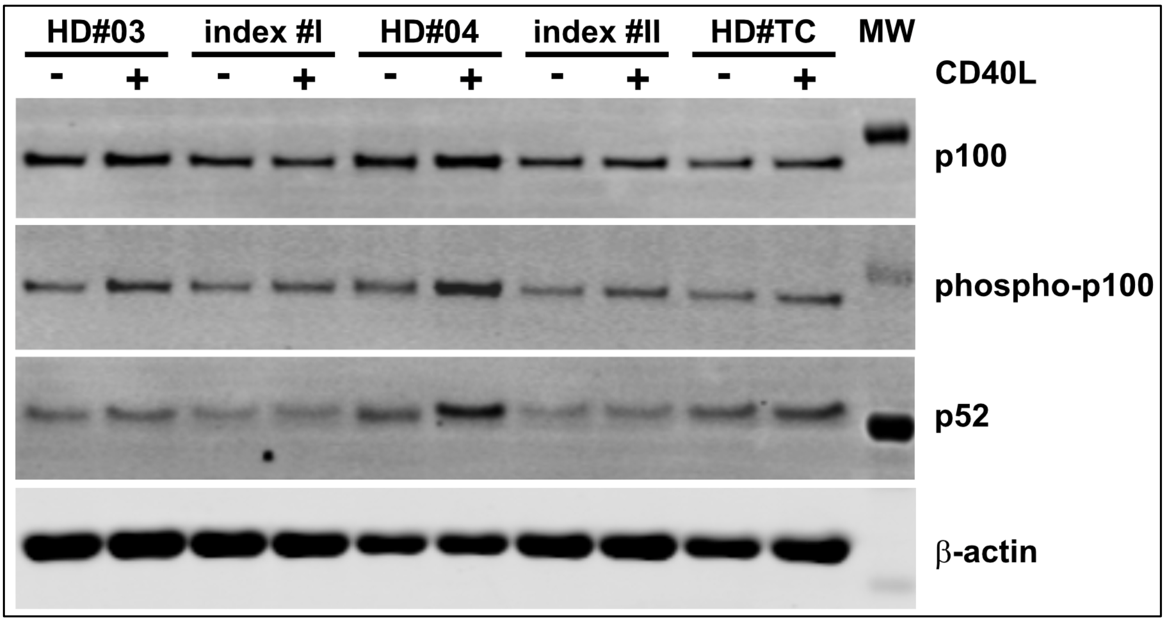

Diminished generation of p52 in CD40-ligand stimulated patient-derived EBV-immortalized B cells.

Cells were stimulated with 2µg/ml CD40L for 4h and p100, phospho-p100 and p52 levels were determined by Western blotting. Two separately generated EBV B cell lines from the index patient were compared to three healthy donor cultures. Relative band intensities indicate reduced average levels of p52 in patient-derived cells (unstimulated: 43.8 ± 17.7%; stimulated: 57.7 ± 31.5% of HD levels), whereas phospho-p100 (unstimulated: 83.4 ± 36.5%; stimulated: 76.7 ± 26.1% of HD levels) and p100 (unstimulated: 109.5 ± 50.7%; stimulated: 74.6 ± 27.5% of HD levels) were variable.

Supplementary Figure S3

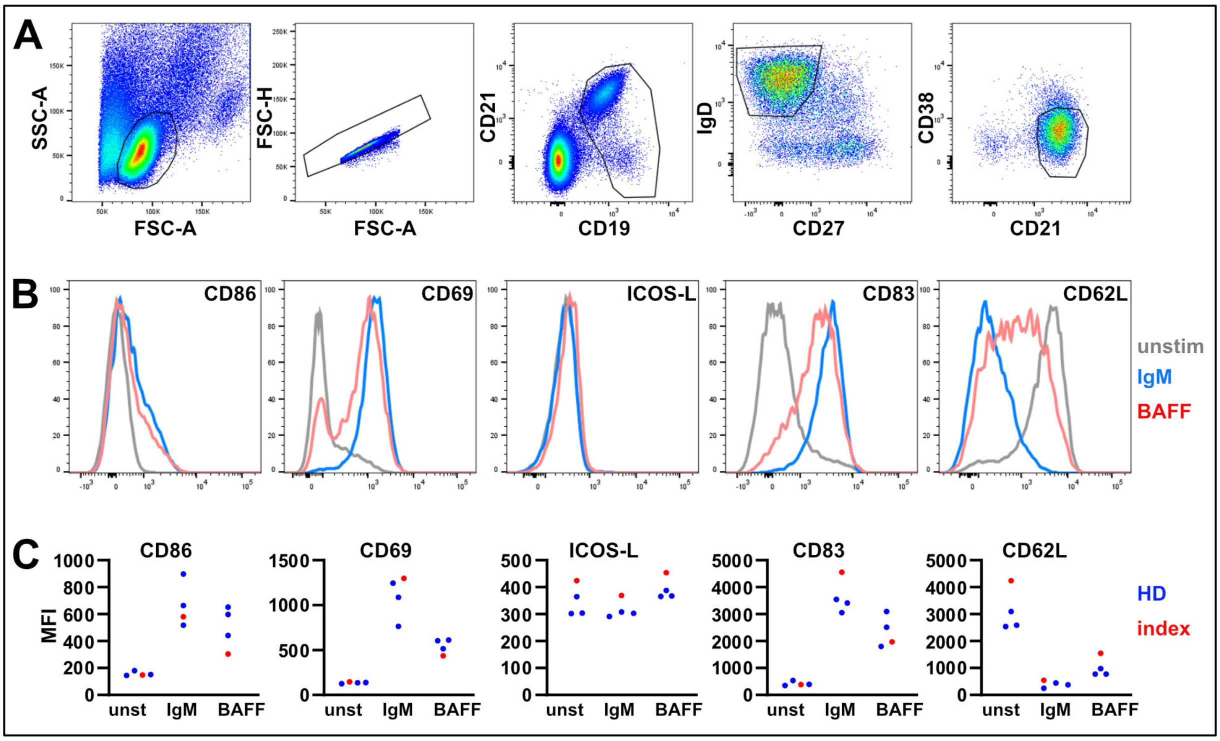

Alterations of surface markers upon activation of the alternative NF-κB signaling pathway in B cells carrying the heterozygous NFKB2 missense variant c.781C>T.

(A) Gating strategy for naïve CD21-positive B cells. (B) Representative histograms for CD86, CD69, ICOS-L, CD83 and in CD62L in CD21-positive B cells either unstimulated or after stimulation of canonical pathway with anti-IgM or of the alternative pathway with rhBAFF. (C) Mean fluorescence intensity (MFI) of the indicated markers in CD21-positive naïve B cells either unstimulated or after stimulation with anti-IgM or rhBAFF in three healthy donors (blue) or the index patient (red).

Supplementary Figure S4

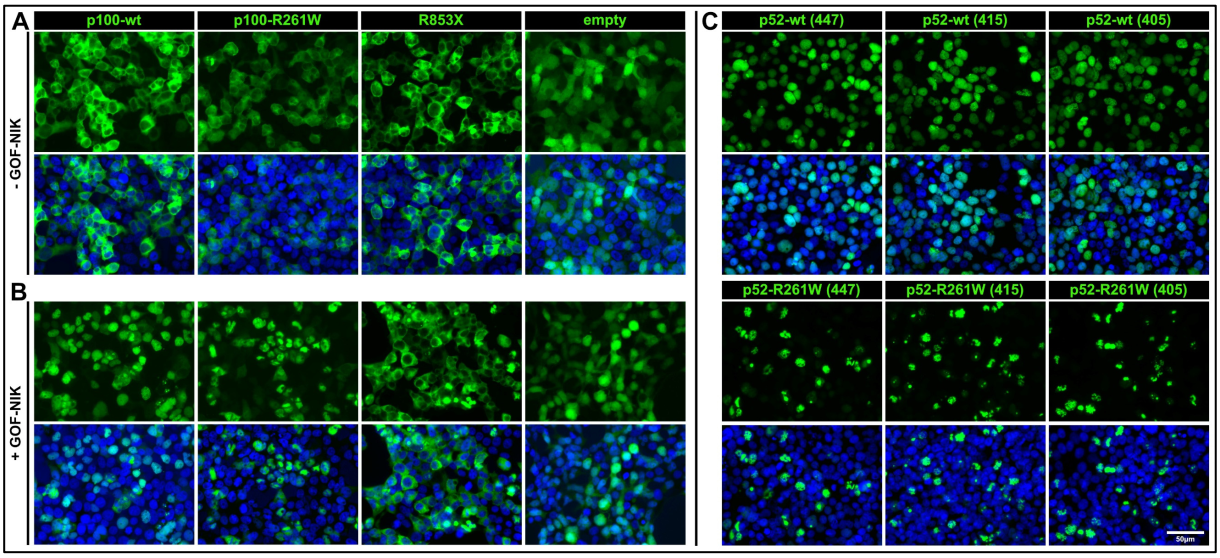

The NF-κB2 missense variant R261W causes sub-nuclear mis-localization of p52.

Fluorescence microscopy of HEK293T cells transiently transfected with the indicated expression vectors as shown in Figure 2. Overlay images are shown with the EGFP (green) and nuclei (Hoechst 33342 staining, blue). (A) EGFP-tagged wildtype, missense-mutant R261W or truncated R853X variants of p100, compared to an empty EGFP-vector control. All three EGFP-p100 proteins localize exclusively to the cytoplasm. The EGFP-p100-R261W gains substantially lower fluorescence intensities (indicating accelerated decay), whereas the truncated R853X yields expression levels indistinguishable from the wildtype control. EGFP alone shows no specific subcellular localization. (B) Same panel as shown in (A) but with co-transfected GOF-NIK to promote p100-to-p52 conversion. The fluorescence signals corresponding to the processed forms of wildtype and R261W-mutant p100 localize to the nuclei in a large proportion of the co-transfected cells. Yet, the EGFP-p52-wt shows a uniform subnuclear distribution while EGFP-p52-R261W accumulates inside the nuclei. The truncated non-processable R853X variant mainly remains within the cytoplasmic compartment. EGFP alone remains unchanged. (C) Differently sized EGFP-tagged wildtype, or missense-mutant R261W variants of p52, corresponding to either amino acids 1-447, 1-415 or 1-405 relative to the precursor. Regardless of the length of the C-terminal extensions, all three ectopically expressed wildtype p52 variants exclusively localize to the nuclei with a homogeneous distribution whereas all three R261W-mutant forms accumulate in high-intense spot-like aggregates inside the nuclei. Please note: the subnuclear pattern of the endogenously generated and the three artificially expressed EGFP-p52-R261W is different, whereas the pattern of all EGFP-p52-wildtype proteins is identical.

Supplementary Figure S5

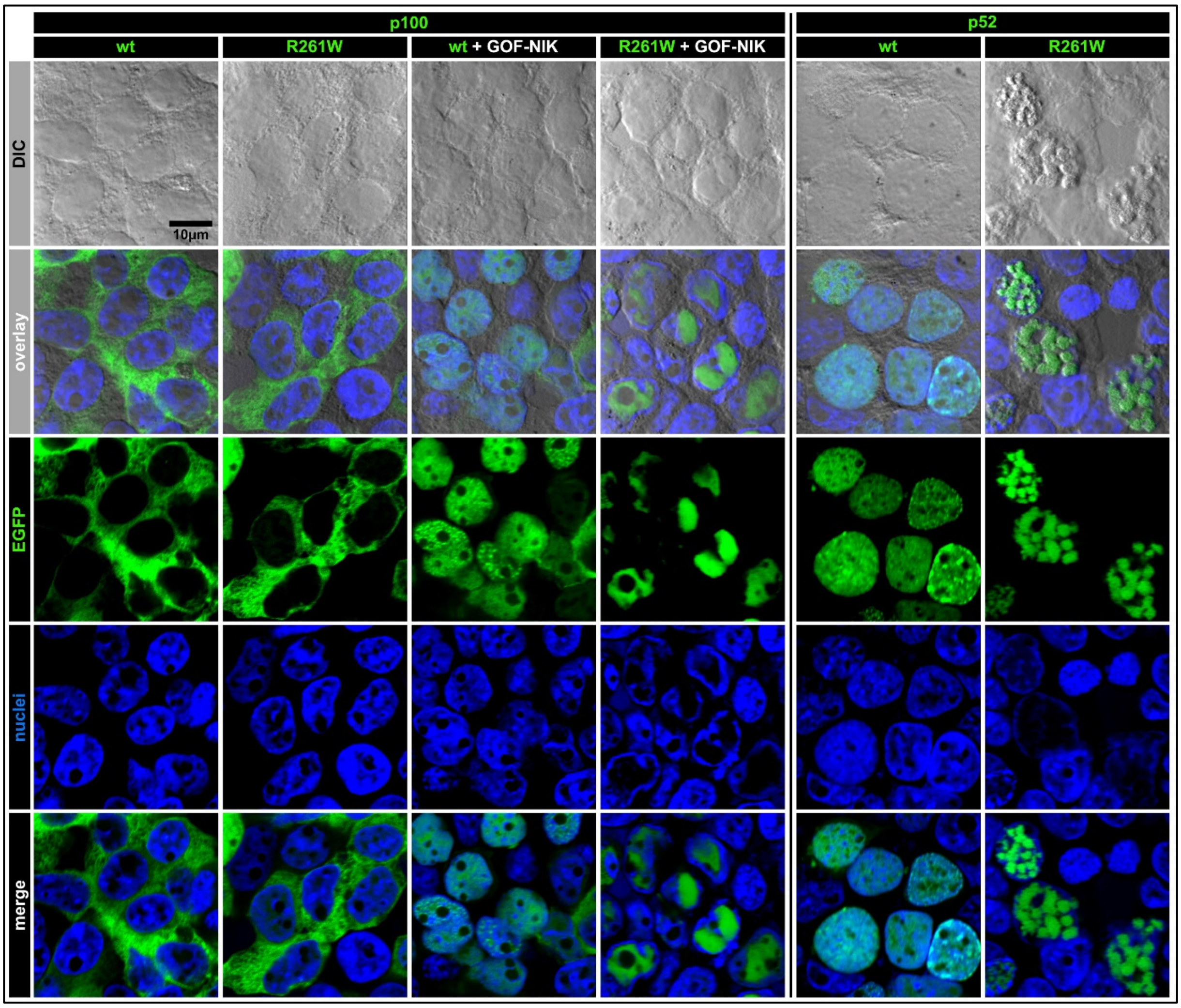

Subnuclear aggregates of R261W missense-mutant p52 proteins displace the genomic DNA and cause morphological aberrations of the nuclei in transfected cells.

HEK293T cells were transiently transfected with the indicated expression vectors as shown in Figure 2 and Supplementary Figure S4 and analyzed by confocal microscopy. DIC and overlay images (upper panels) demonstrate morphological changes of the nuclei with either 'blebbing' (endogenously generated EGFP-p52-R261W *via* GOF-NIK-enforced precursor processing) and 'granules' (artificial overload of the nuclei with immediately expressed EGFP-p52-R261W). Please note: both effects represent experimental artifacts due to the massive overexpression, which does not reflect a physiological situation e.g. as in patient-derived cells.

Supplementary Figure S6

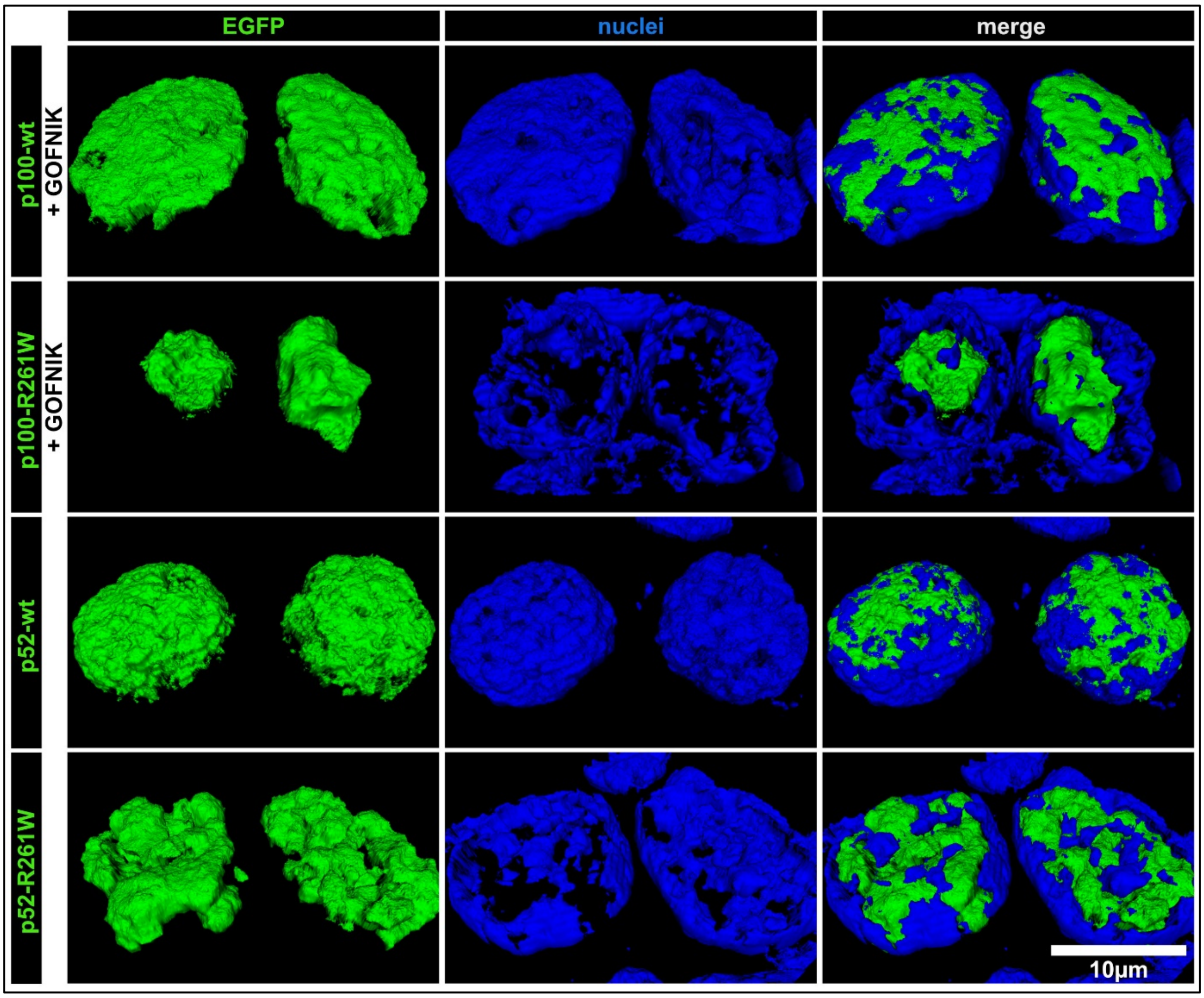

Subnuclear deposition of overexpressed EGFP-p52-R261W into dense aggregates.

3D re-constructed confocal Z-stack images of nuclei from HEK293T cells transiently transfected with the indicated expression vectors as shown in Figure 2 and Supplementary Figure S4 and S5. Both, endogenously generated and ectopically expressed wildtype EGFP-p52, co-localizes with the genomic DNA within the nuclei. In contrast, both, the endogenously generated and the ectopically expressed missense mutant EGFP-p52-R261W, cause displacement of the genomic DNA within the nuclei due to the formation of a single huge central protein aggregate or a diffuse assembly of various smaller aggregates, respectively.

Supplementary Figure S7

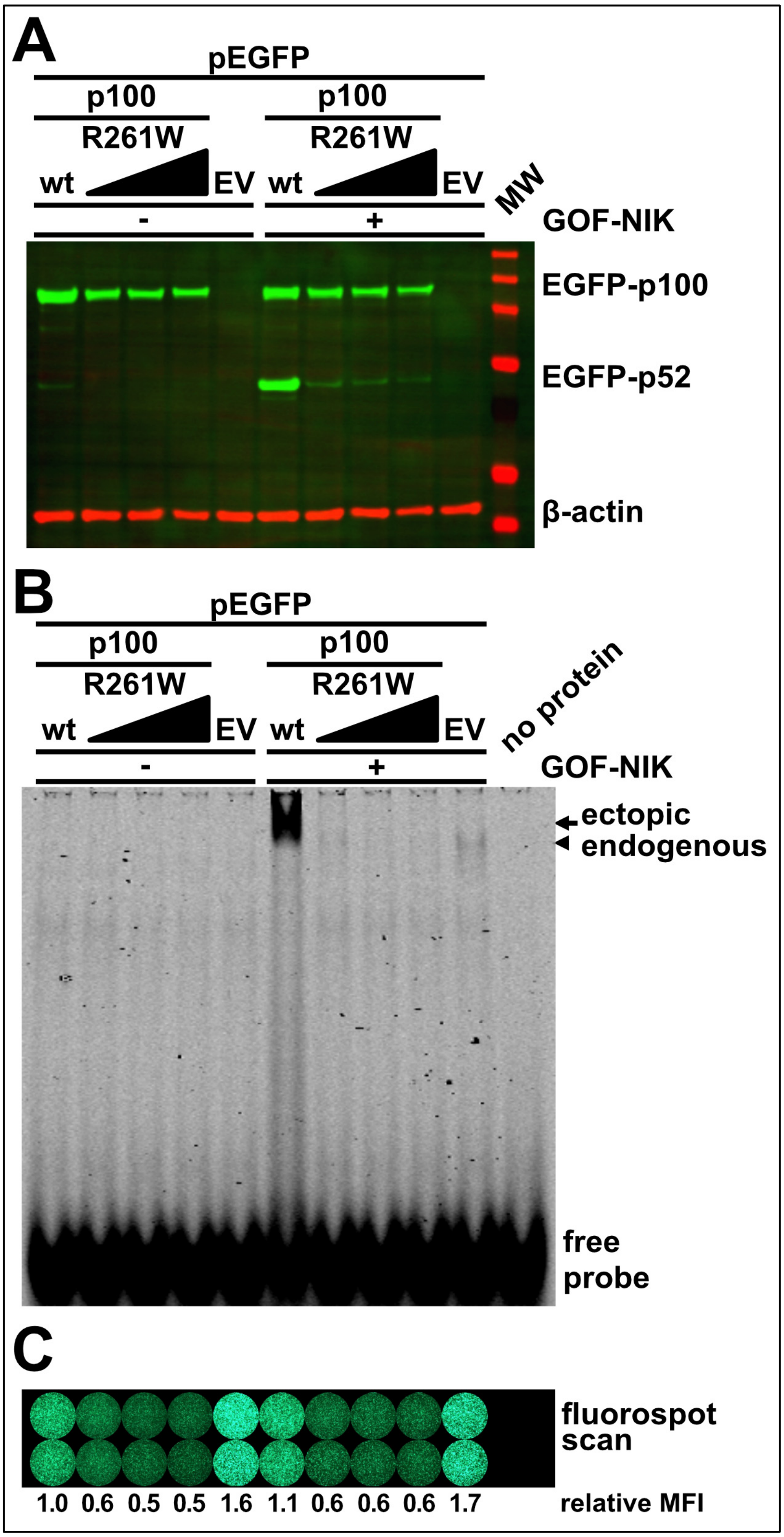

The missense mutant p100-R261W precursor gains limited overexpression levels, while generation of the processed subunit p52 is severely impaired.

HEK293T cells were transiently transfected in duplicates with increasing amounts of plasmid DNA (300/600/900 ng per well in 48-well format) encoding a N-terminally EGFP-tagged derivate of the mutant p100-R261W to achieve saturated conditions with the highest possible protein expression. The wildtype p100 (300ng) was included as a positive control for efficient precursor processing. (A) Crude cell extracts (transfection sample I each) were analyzed by Western blotting for expression of the p100 precursor proteins and the presence of their processing products, the shorter p52 transcription factor subunits. (left) Weak constitutive p100-to-p52 conversion is only observed with the EGFP-fused wildtype p100. The expression level of EGFP-p100-R261W already reached its maximum with the lowest tested DNA amount. (right) Co-delivery of a constitutively active variant of the upstream kinase NIK (GOF-NIK; 12.5 ng each) massively enhances the amount of the processed EGFP-fused wildtype p52 but not of the mutant p52-R261W. (B) Identical transfection experiment as shown in (A). EMSA using nuclear protein extracts from transfected cells (transfection sample II each) demonstrates robust generation of DNA-binding activity only after GOF-NIK-enhanced conversion of the EGFP-fused wildtype p100 into nuclear EGFP-p52-wt. The endogenously generated EGFP-p52-R261W does not produce DNA-binding activity, although GOF-NIK promotes nuclear DNA-binding activity of endogenous NF-κB proteins (faint band in the EV empty vector control). (C) Scan of the duplicate transfection samples in 48well format using automated fluorescence microscopy prior to cell harvest. Relative fluorescence intensities (MFI) normalized to the leftmost EGFP-p100wt samples are indicated below.

Supplementary Figure S8

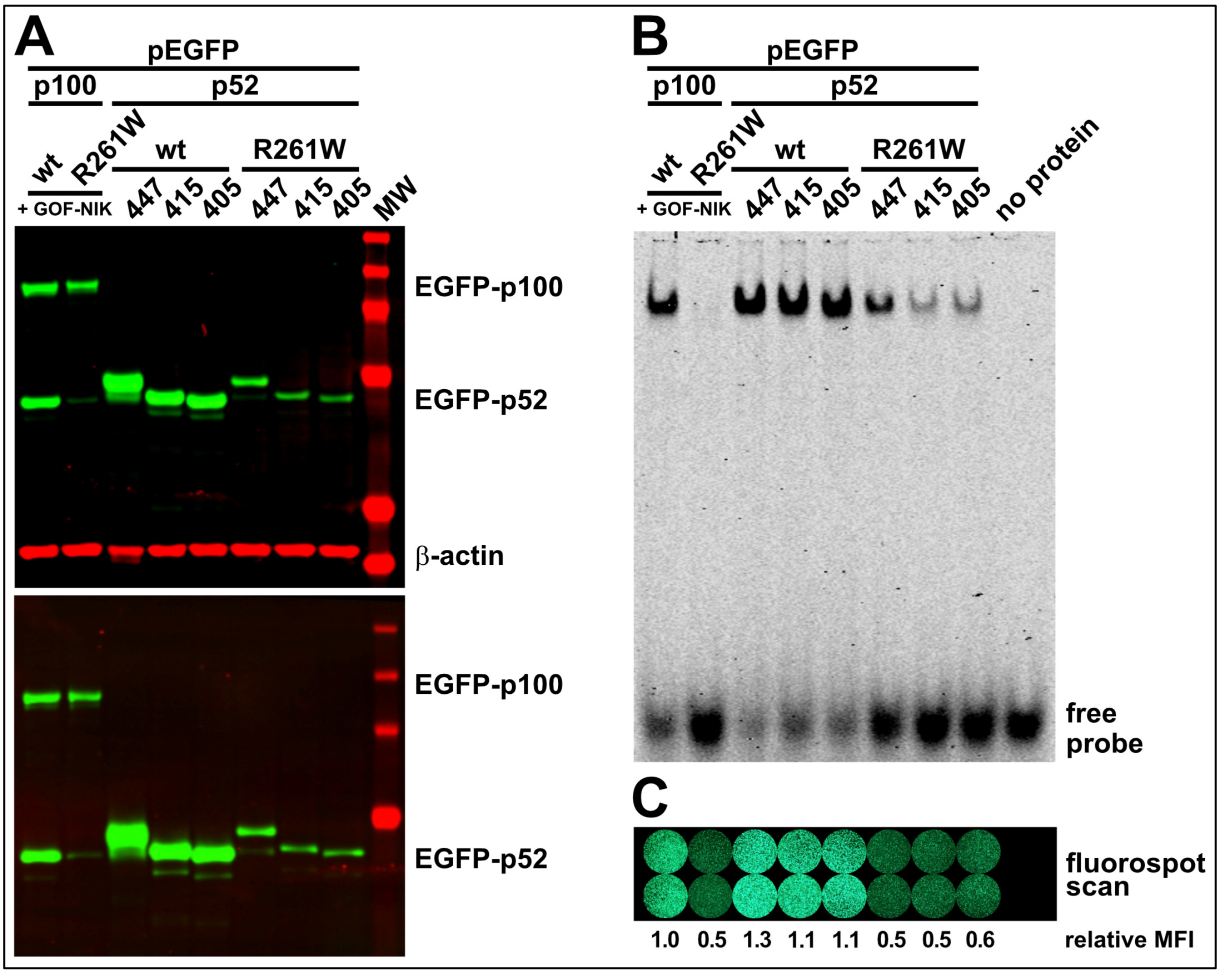

The R261W missense change causes severe protein-instability of and the mature transcription factor subunit p52.

HEK293T cells were transfected with wildtype and R261W mutant EGFP-p100 together with GOF-NIK to promote p100-to-p52 conversion by endogenous mechanisms. In addition, three differently sized wildtype or R261W-mutant EGFP-p52 variants (447, 415 or 405 amino acid each) were transfected in parallel to test whether the C-terminal extensions influence the deleterious effect caused by the missense change. (A) Western blotting confirms efficient processing only of the wildtype EGFP-p100 whereas expression and processing of EGFP-p100-R261W is limited. Expression levels of all three ectopic R261W-mutant p52 forms are severely reduced compared to their wildtype counterparts (with the 447aa mutant protein gaining 21.2 ± 5.5% of the wildtype levels; 415aa: 10.0 ± 2.4%; 405aa: 9.5 ± 0.5%). The endogenously generated p52 is smaller than the 447 amino acid form, but its precise size could not be determined. Although the longer 447 amino acid form gains higher expression levels, the protein-decaying defect caused by the R261W missense variant occurs in all p52 protein forms, independent from the C-terminal extensions. (B) The levels of the NF-κB-dependent

DNA-binding activities detectable in nuclear extracts of transfected cells by EMSA correspond to the protein expression levels analyzed in (A). (C) Fluorescence microscopic scan of the duplicate transfection samples in 48-well format prior to cell harvest for Western blotting shown in (A) and EMSA shown in (B). Numbers indicate relative fluorescence intensities (MFI) normalized to the EGFP-p100wt samples. In five independent experiments the 447aa mutant protein yielded (0.43±0.05)-fold, the 415aa mutant protein (0.44±0.05)-fold and the 405aa mutant protein (0.38±0.05)-fold intensities compared to their wildtype counterparts.
